# Single-cell transcriptomics reveals that glial cells integrate homeostatic and circadian processes to drive sleep–wake cycles

**DOI:** 10.1038/s41593-023-01549-4

**Published:** 2024-01-23

**Authors:** Joana Dopp, Antonio Ortega, Kristofer Davie, Suresh Poovathingal, El-Sayed Baz, Sha Liu

**Affiliations:** 1 Center for Brain & Disease Research, VIB-KU Leuven, Leuven, Belgium; 2 Department of Neurosciences, KU Leuven, Leuven, Belgium; 3 Leuven Brain Institute, KU Leuven, Leuven, Belgium; 4 Zoology Department, Faculty of Science, Suez Canal University, Ismailia, Egypt

**Keywords:** Sleep, Circadian regulation, Glial biology

## Abstract

The sleep–wake cycle is determined by circadian and sleep homeostatic processes. However, the molecular impact of these processes and their interaction in different brain cell populations are unknown. To fill this gap, we profiled the single-cell transcriptome of adult *Drosophila* brains across the sleep–wake cycle and four circadian times. We show cell type-specific transcriptomic changes, with glia displaying the largest variation. Glia are also among the few cell types whose gene expression correlates with both sleep homeostat and circadian clock. The sleep–wake cycle and sleep drive level affect the expression of clock gene regulators in glia, and disrupting clock genes specifically in glia impairs homeostatic sleep rebound after sleep deprivation. These findings provide a comprehensive view of the effects of sleep homeostatic and circadian processes on distinct cell types in an entire animal brain and reveal glia as an interaction site of these two processes to determine sleep–wake dynamics.

## Abstract (web summary)

Dopp et al. profiled gene expression in single cells from the whole fly brain, revealing how it changes with sleep/wakefulness states and circadian times. The findings highlight the role of glia in integrating sleep drive and circadian processes.

## Main

Sleep is regulated by two independent processes: the circadian system and the sleep homeostatic system^1^. The circadian clock primarily regulates the timing of sleep, known as process C. It consists of a transcriptional–translational feedback loop of core clock genes^2^. At the cellular level, our current understanding of the circadian timing system focuses on the circadian pacemaker regions and neurons, such as the suprachiasmatic nucleus (SCN) in mammals^3^ and 150 clock neurons in *Drosophila*^4^. However, it is unclear whether and how process C affects the transcriptomes of any given brain cell population apart from pacemaker regions and neurons.

The sleep homeostat monitors the sleep need that accumulates with the amount of time that an animal has been awake to determine the sleep drive, known as process S. Our understanding of the nature of the sleep homeostat is limited. The effects of the sleep–wake cycle and sleep homeostasis on the transcriptome have been studied in bulk brain tissue samples^5^ or bulk synaptosomes^6^ in mammals and whole-brain tissue in flies^7,8^. The results of these studies were inconsistent, possibly because of averaging transcriptomic changes of heterogeneous cell populations (Supplementary Fig. 1). Recently, single-cell RNA sequencing (scRNA-seq) was applied to study the transcriptional changes during sleep homeostasis in mice^9^. However, these studies only focused on certain regions of the brain; a comprehensive and unbiased understanding of sleep/wakefulness and sleep homeostasis-associated transcriptomic changes across cell populations of an entire brain is lacking.

In this study, we sampled adult *Drosophila* brains at different sleep, wakefulness and sleep pressure states at different circadian times, and performed scRNA-seq, thereby creating a comprehensive transcriptional atlas of the sleeping animal brain (see https://www.flysleeplab.com/scsleepbrain for the single-cell gene expression atlas and analyses links). We found that sleep/wakefulness states, sleep homeostasis and circadian rhythm have different transcriptional correlates depending on cell identity. Our data also suggest that gene expression in most cell populations correlates either with process C or process S, with the exception of glial cells, which instead are affected by both processes simultaneously. We propose a model whereby homeostatic and circadian processes directly interact in glial cells to regulate sleep.

## Results

### Single-cell transcriptomes of sleep–wake and circadian times

**Fig. 1:**
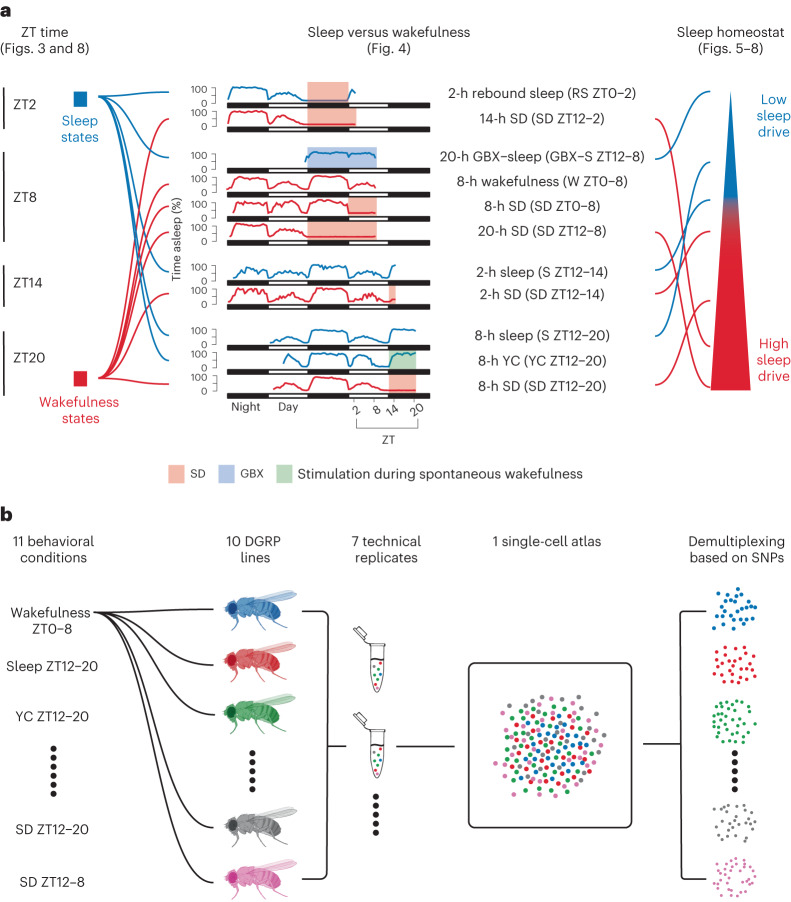
Sampling flies at sleep and wakefulness states for subsequent transcriptional profiling. **a**, Flies were sampled at four different ZT times and 11 different sleep or wakefulness states. Seven of the conditions were ordered according to accumulated sleep pressure. The corresponding downstream analyses of each of the three correlates are described in the corresponding figure (displayed in parentheses). **b**, In each of seven technical replicates (runs), each condition was linked to two or three DGRP lines. The link between condition and DGRP line was changed in every run. The flies’ central brains were dissected and the tissue was dissociated in a single tube, minimizing batch effects. Conditions were separated by demultiplexing the sequenced reads based on unique SNPs of DGRP lines. Source data

**Extended Data Fig. 1:**
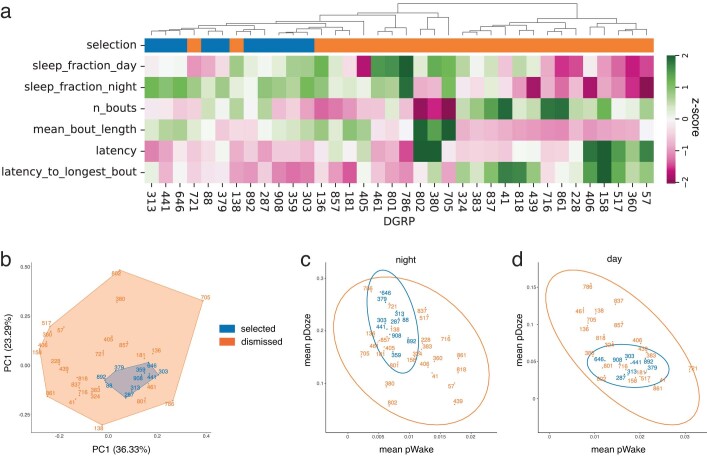
Sleep behavior screening of 36 DGRP lines to select suitable DGRP lines for single cell transcriptomics. **a**. Clustered heatmap of quantified sleep amount during day and night, sleep bout length and number, latency to first and longest sleep bout during nights across all tested DGRP lines. Based on these parameters ten DGRP lines were selected (n = 355, yellow) and 26 dismissed (n = 425, purple). **b**. Principal Component Analysis on mean and standard deviation per sleep parameter and DGRP line. **c-d**. Scatterplot showing the probability to switch from a wake to a sleep state (pDoze) or vice versa (pWake) averaged across flies of the same DGRP line. Source data

**Extended Data Fig. 2:**
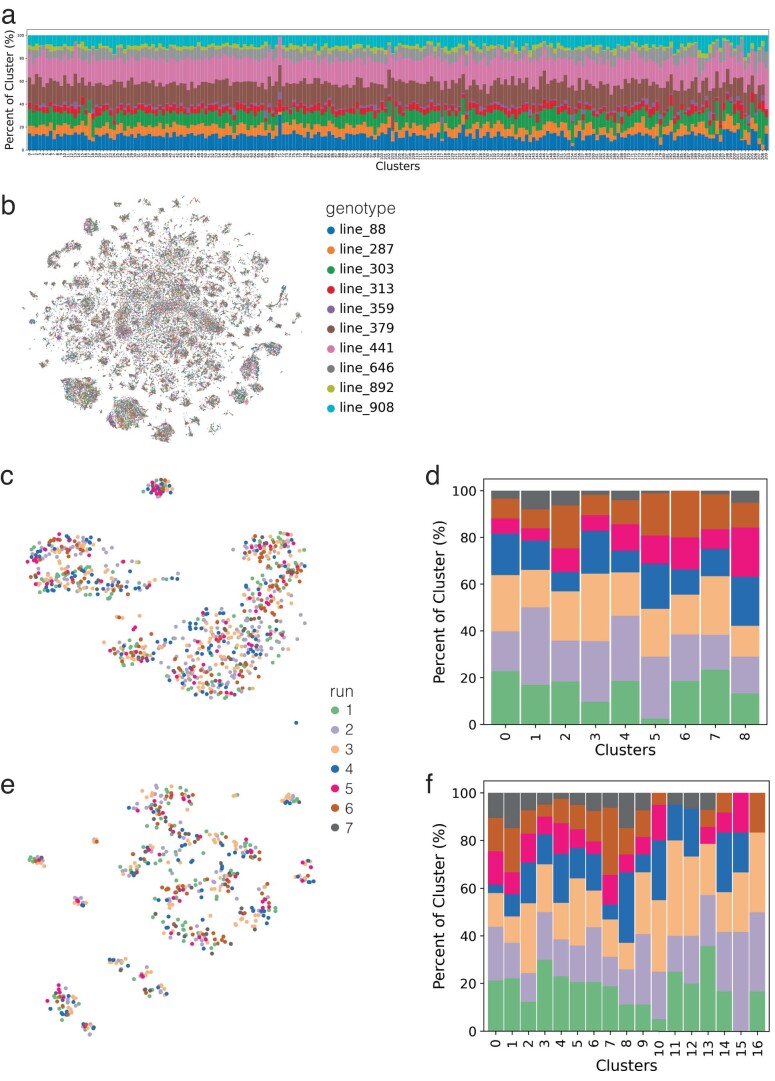
Bias analysis of (sub)clusters. **a**. Normalized number of cells coloured by genoype for each cluster in resolution 8. Most clusters have a relatively similar number of cells from each genotype. **b**. tSNE of all cells coloured by genotype shows cells of different DGRP lines mix well. **c, e**. tSNE displaying cells colored by assignment to their run reveals that there are no technical batch effects for EB ring (**c**) and clock neuron (**e**) subclustering. **d, f**. Proportion of cells in each cluster colored by their assignment to their run for EB ring (**d**) and clock neuron (**f**) subclusters.

We performed scRNA-seq with 10x droplet microfluidics on adult *Drosophila* central brains (without optic lobes), sampled at distinct points of the sleep–wake cycle across different circadian times (Fig. 1a). These sample points can be (1) grouped according to four Zeitgeber (ZT) times to analyze the transcriptional correlates of circadian rhythms; (2) grouped by ‘sleep’ and ‘wakefulness’ states according to the animal’s vigilance status at the time of sample collection to examine sleep/wakefulness correlates; and (3) selected and ordered according to the fly’s level of sleep drive to determine the molecular changes associated with the sleep homeostat. To minimize the technical batch effects that may mask true biological responses, we applied demultiplexing based on natural variation between wild-type genotypes^10^. Specifically, instead of associating each batch to a different sleep or wakefulness state, we associated a different *Drosophila* genetic reference panel (DGRP)^11^ line to each behavioral condition (Fig. 1b and Supplementary Table 1). The full genome of all DGRP lines has been sequenced, thus they can be distinguished from one another according to their unique single-nucleotide polymorphisms (SNPs). Thanks to this natural genetic variation, RNA-seq data disclose the associated sleep or wakefulness states, which allows the pooling of brains from different behavioral conditions in a single batch (Fig. 1b and Methods). In addition to minimizing technical batch effects, we also counteracted potential genotype-specific effects by repeating the same conditions with different DGRP lines with similar sleep profiles in subsequent batches (Supplementary Table 1). To find the DGRP lines with the most similar sleep behavior, we carefully chose them by (1) preselecting 36 DGRP lines based on sleep architecture metrics previously reported in a large collection of DGRP lines^12^ and (2) thoroughly screening those 36 lines across several sleep parameters. The criteria for selection were (1) a robust amount of consolidated nighttime sleep and a low amount of daytime sleep, and (2) low between and within-genotype variability (Extended Data Fig. 1a). Principal component analysis (PCA) of these sleep parameters showed that the ten selected lines grouped together closer than the discarded lines (Extended Data Fig. 1b). Similar clustering of selected versus discarded lines was also visible when plotting the probabilities to transition from a wakefulness to a sleep state (pDoze) or vice versa (pWake), which indirectly assessed sleep drive and sleep depth in *Drosophila*^13^ (Extended Data Fig. 1c,d). The low variability between the sleep phenotype of the selected ten DGRP lines allowed us to repeat each condition with several lines in the same and in different batches (Supplementary Table 1). The success of this strategy is attested by the homogenous distribution of cells from different genotypes (Extended Data Fig. 2a), which blended within cell subtypes even without applying batch integration algorithms (Extended Data Fig. 2b).

**Fig. 2:**
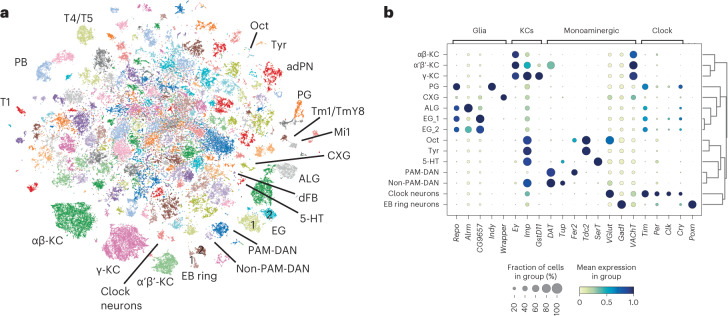
Cell-type annotations in a single-cell atlas of the sleeping fruit fly. **a**, *t*-Distributed stochastic neighbor embedding (*t*-SNE) plot of the entire dataset of 106,762 single cells with annotated clusters and expression of key marker genes of glial and neuronal cell types used to annotate the clusters. **b**, Marker gene expression across most of the clusters annotated in **a**. 5-HT, 5-hydroxytryptamine (serotonergic) neuron; adPN, anterodorsal projection neuron; ALG, astrocyte-like glia; CXG, cortex glia; EG, ensheathing glia; PB, protocerebral bridge neuron; PG, perineurial glia.

**Extended Data Fig. 3:**
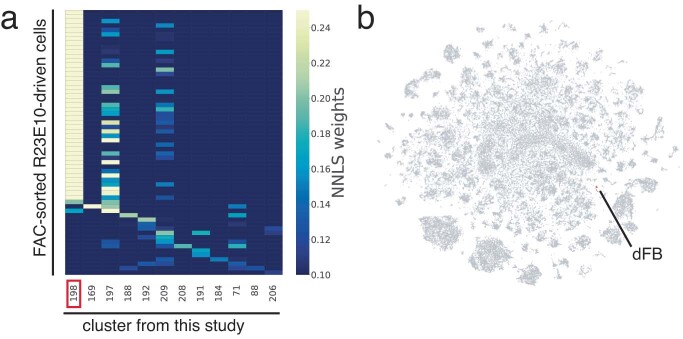
Annotation of dFB cluster. **a**. Heatmap of those 12 clusters of our 10x data that map to at least one R23E10-Gal4 FAC-sorted cell from the publicly available scRNA-seq data by Davie et al.^14^. Cluster 198 from this study shows the highest correlation for the majority of sorted cells, indicating a positive match for this cluster with dFB cells. Similarity between FAC-sorted data and clusters of this study was calculated with a non-negative least-squares regression model (NNLS) (Stanescu et al., 2017). **b**. Annotation of cluster 198 as dFB in tSNE of all cell types.

To obtain an overview of the captured cell types, we first performed dimensionality reduction on all 106,762 cells combined and identified 214 clusters of cells (Fig. 2a). We annotated cells based on previously used marker genes in the fly brain cell atlas^14,15^, allowing us to successfully assign 22,988 cells to one of 25 known cell types (21.5%) (Fig. 2b and Supplementary Table 2), including five glial subtypes, Kenyon cells (KCs), clock neurons and cell types containing known sleep/wakefulness regulating circuits such as, non-protocerebral anterior medial (PAM) dopaminergic neurons (DANs)^16^, tyraminergic (Tyr) and octopaminergic (Oct) neurons^17,18^, and ellipsoid body (EB) ring neurons^19,20^. Another cell type involved in sleep^21^, the dorsal fan-shaped body (dFB) neuron, was annotated by correlating the previously published transcriptome of fluorescence-activated cell (FAC)-sorted dFB neurons^14^ with our gene expression data (Extended Data Fig. 3).

### Cycling of core circadian genes in clock neurons and glia

**Fig. 3:**
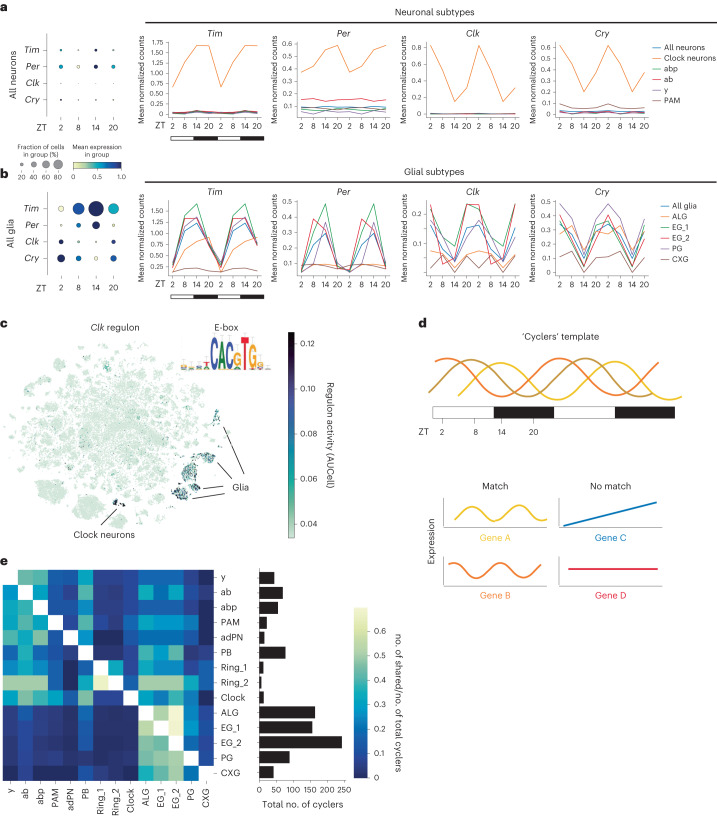
Oscillating transcripts in neurons and glia. **a**,**b**, Circadian expression levels of the core clock genes *per*, *tim*, *cry* and *Clk* averaged across all neurons, some neuronal subtypes (**a**) and all glia and glial subtypes (**b**). The size of the dot indicates the fraction of cells in each group. Mean expression in each group was normalized to gene expression across the four ZT time points. The data in the line plot were plotted twice to better visualize the cycling patterns. **c**, *Clk* regulon and its activity across all cell types. **d**, Schematic of the template to detect cycling transcripts based on Borbély’s two-process model^1^. Examples of genes whose expression would match (yellow and orange) or not match (blue and red) the template are given. **e**, Heatmap of intersecting cycling genes between all annotated cell types with at least one shared gene relative to the total number of cycling genes (at least five genes, right bar plot) according to cell type. Genes with a JTK cycle Benjamini–Hochberg-corrected *P* < 0.05 were considered as significantly cycling.

**Extended Data Fig. 4:**
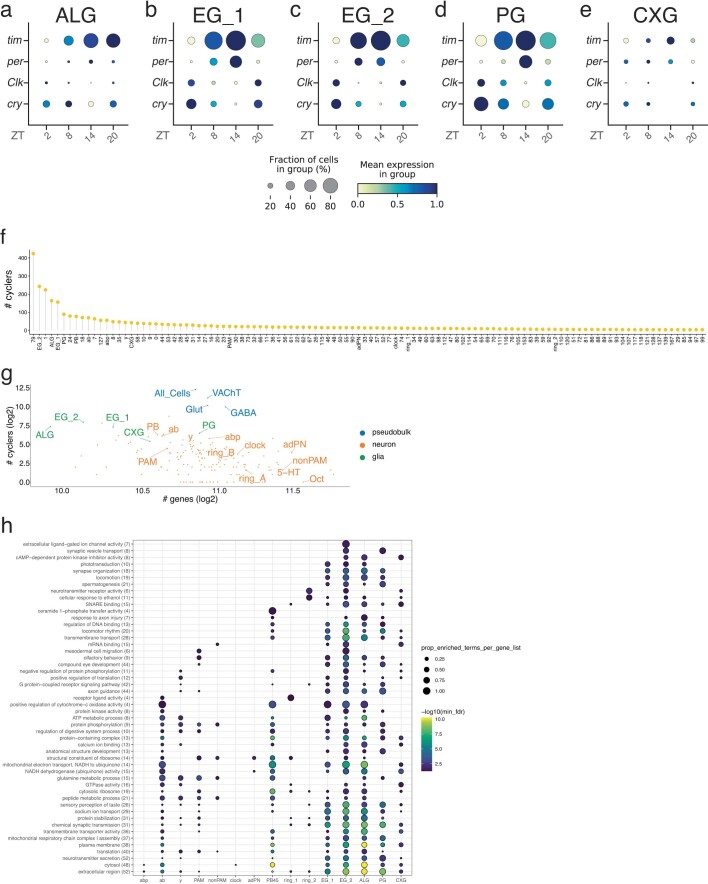
Oscillation of core circadian genes in glia and number and gene ontology of cyclers across cell types. **a-e**. Circadian expression levels of core clock genes *per, tim, cry* and *Clk* for each glial subtype. **f** Number of cyclers across all clusters with at least three correlates. **g**. Correlation between number of genes and number of cyclers for pseudobulk samples, neuronal and glial cell types. **h**. Gene ontology parent terms enriched across annotated clusters. Number of child terms per parent term in brackets. Size of dot indicates fraction of child terms associated with the parent term.

The expression levels of circadian clock gene transcripts cycle in a daily manner in clock neurons^2^. To validate our single-cell transcriptomic dataset, we asked whether the data accurately captured the cycling expression of core clock genes between the four sampled ZT time points. Indeed, as reported previously in clock neurons^22^, *period* (*per*) and *timeless* (*tim*) transcripts were expressed at higher levels in the early night compared to the early day, while the opposite applied to *cryptochrome* (*cry*) and *Clock* (*Clk*) mRNA (Fig. 3a). Beyond validating our dataset, we examined whether and how the clock genes were cycling in all remaining cell populations. In mammals, cycling of clock gene expression in cell populations other than the SCN has been reported, for example, in cortical regions^23^. Interestingly, we report that the expression and cycling of these genes is restricted specifically to clock neurons and glial cells (Fig. 3a,b and Extended Data Fig. 4a–e); this is consistent with previous immunostaining results for clock proteins in the fly brain^24,25^. To further confirm this, we tested the activity of the *Clk* regulatory network (regulon) across all cells by applying SCENIC^26^. This regulon was defined by the previously identified *Clk* binding element E-box sequence and its target genes, including *tim*, *cry* and *vrille* (*vri*)^27^. Our data show that the *Clk* regulon is only active in clock neurons and most glial subtypes, but not in other neurons (Fig. 3c), further indicating that core clock genes are expressed and cycle specifically in *Drosophila* clock neurons and glia. Interestingly, while *Clk* expression is restricted to the clock neurons and glia, other core clock genes, *per*, *tim* and *Cycle (Cyc)* are expressed across more cell types. The finding that no cell type expresses *Clk* without expression of other core circadian genes is consistent with the notion that *Clk* is a circadian master regulator^28^.

Fly glia were previously associated with the expression of clock genes, especially astrocyte-like glia (ALGs)^25^ and perineurial glia (PG)^29^. Interestingly, our data showed that the molecular clock runs with different phases depending on the cell type. Specifically, the expression of *tim* and *per* mRNA is high throughout the night in clock neurons, while in PG and ensheathing glia (EG), its expression was already decreased at ZT20 (Fig. 3b and Extended Data Fig. 4a–e). In contrast, *tim* expression in astrocytes only peaks at ZT20. The delayed clock in astrocytes compared to neurons is also observed in the mouse SCN^30^. Taken together, the expression of key clock genes in glia in addition to clock neurons, suggests that these cells are directly involved in circadian regulation of rhythmic behaviors, including sleep.

### Cyclers are more enriched in glia than neurons

Next, we comprehensively identified all transcripts (cyclers) oscillating in all cell types by applying the JTK cycle algorithm (Fig. 3d, Supplementary Table 3 and Methods)^31^. While the expression of the molecular clock was restricted to clock neurons and glial cells, most cell clusters (82.5%) had at least one cycler (Extended Data Fig. 4f) and 14 of the 19 annotated clusters had at least five cyclers (Fig. 3e). Like the analysis of core clock genes, glial cells stand out, this time by showing the highest number of cyclers, especially considering that they typically express fewer genes than neurons (Extended Data Fig. 4g). When comparing the overlap of cyclers between all cell types, we found that only between 20% and a maximum of 50% of cyclers are shared. We identified a higher number of shared cyclers between closely related cell types, such as the three KC subtypes, and particularly between neuropil-associated glial subtypes (ALGs and EGs) (Fig. 3e). Similarly, gene ontology (GO) analysis of cyclers showed that glial subtypes shared more similar signaling pathways compared to neuronal subtypes (Extended Data Fig. 4h). GO terms encompassed ‘mitochondrial electron transport, NADH to ubiquinone’, ‘sodium ion transport’, ‘chemical synaptic transmission’ and ‘extracellular region’, suggesting that many key functions of glia including metabolism, ionic homeostasis and gliotransmission, are affected by the circadian clock. In summary, these data suggest that different cell populations respond differently to process C, with glial cells being the most affected.

### Sleep/wakefulness correlates differ between cell populations

**Fig. 4:**
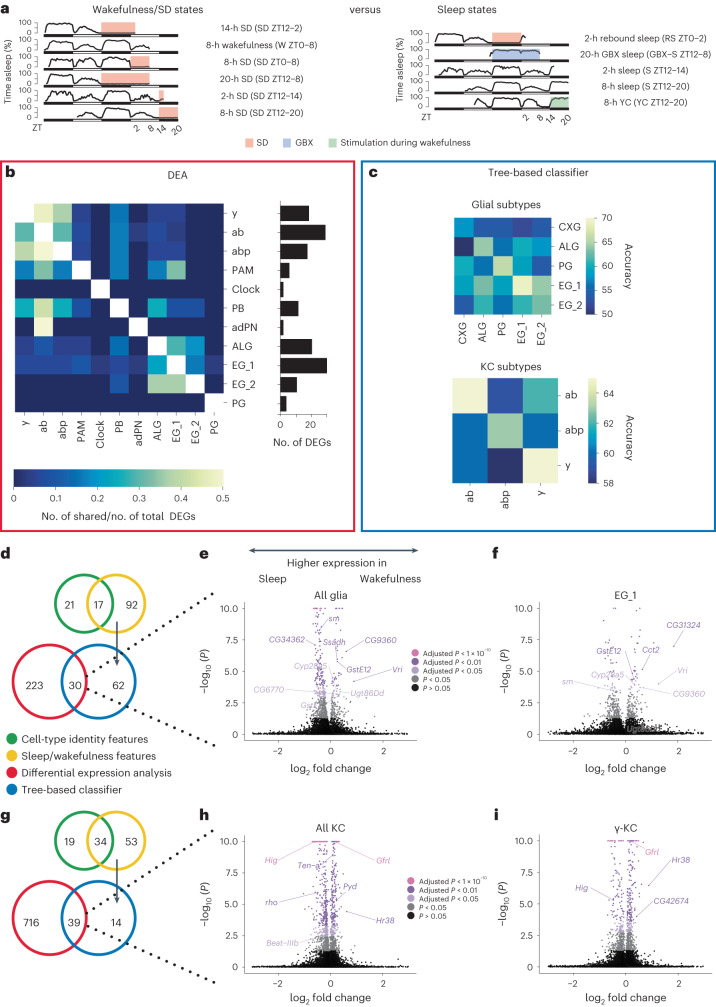
The transcriptomes of glia and KCs change differently between sleep and wakefulness. **a**, Grouping of wakefulness–SD states and sleep states to compare them in differential expression analysis and a tree-based classifier. **b**, Heatmap of intersecting DEGs between all annotated cell types with at least one shared gene relative to the total number of DEGs according to cluster. The bottom bar plot shows the amount of total DEGs with an adjusted *P* < 0.05 and log fold change lower than −0.5 or greater than 0.5. The statistical method used was a Wilcoxon rank-sum test. *P* values were adjusted for multiple-comparisons testing using the Benjamini–Hochberg method. **c**, Classifying glial (top) and KC (bottom) subtypes into sleep or wakefulness labels revealed that performance was highest for the same subtype. **d**–**i**, For glia (**d**) and KCs (**g**), candidate genes were filtered by (1) removing the cell-type features from the sleep/wake features identified using separately trained Explainable Boosting Machines (EBMs) and (2) overlapping significant differential expression analysis (DEA) and EBM results. The volcano plots highlight a selection of the common genes between the two methods for all glia (**e**), EG_1 (**f**), all KC (**h**) and y-KC (**i**).

**Extended Data Fig. 5:**
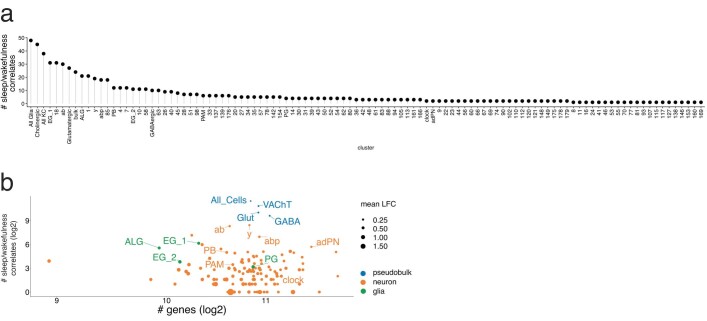
Sleep/wakefulness correlates across all clusters. **a**. Number of sleep/wakefulness correlates across all clusters with at least one correlate. **b**. Correlation between number of genes and number of sleep/wakefulness correlates for pseudobulk samples, neuronal and glial cell types. Dot size indicates average log2 fold change (LFC) across all significant sleep/wakefulness correlates for the respective cluster.

Next, we asked whether we could detect transcript level changes across sleep/wakefulness states (referred to as sleep/wakefulness correlates), similarly to ZT times. We assigned each condition to a sleep or wakefulness group based on whether the animal had consolidated sleep or wakefulness before sampling (Figs. 1a and 4a and Methods). The sleep group included spontaneous sleep, recovery sleep after sleep deprivation (SD), yoked control (YC) and gaboxadol (GBX)-induced sleep^32^. We grouped spontaneous wakefulness and forced wakefulness (SD) to regress out the unwanted effects of ZT time and the potential stress induced by mechanical SD. Then, we performed differential expression analysis between the two groups for each cell population separately and asked whether transcriptomic changes between sleep and wakefulness states differ between distinct cell populations. We found that 46.7% of all clusters and 57.9% of annotated clusters had sleep/wakefulness correlates (Fig. 4b, Extended Data Fig. 5a,b and Supplementary Table 4). KCs and glia had the highest number of differentially expressed genes (DEGs) among the annotated cell types. Considering that glia express the lowest number of genes among all cell types (Extended Data Fig. 5b), these cells may be even more affected by sleep/wakefulness relative to their total expressed genes compared to neurons. Interestingly, most identified DEGs in each of the annotated cell types were unique to that cell type (Fig. 4b). On average only 12.8% or 13.5% of DEGs were shared within neuronal or glial subtypes, respectively. Like cyclers, the overlap of sleep/wakefulness-correlated transcripts was even smaller between neurons and glia, averaging between 3% and 4% only, with the exception of PAM DANs and protocerebral bridge (PB) neurons, which shared between 17% and 33% of their sleep/wakefulness-correlated genes with ALG and EG_1. This low overlap between closely related cell subtypes suggests that sleep–wake cycles affect their transcriptome in unique ways.

**Extended Data Fig. 6:**
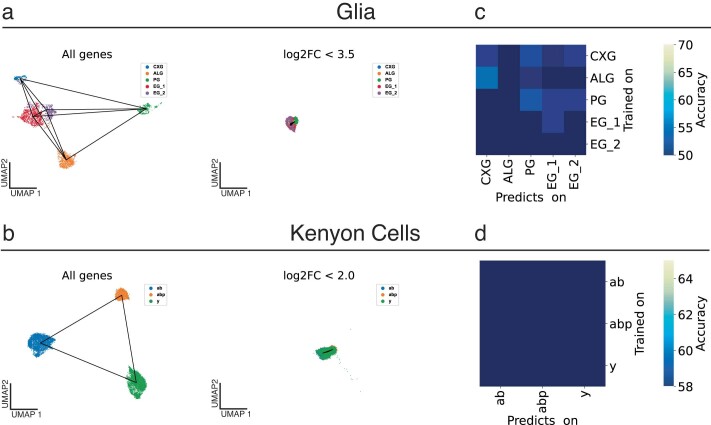
Controlling sleep/wake state classifier by removing marker genes between subtypes of KC or glia. **a, b**. Glial and KC subtypes move closer to each other in a UMAP space, because marker genes above certain log foldchange thresholds are removed. Genes falling below a threshold of 3.5 or 2 for glia and KC, respectively were excluded for training the tree-based EBM classifier. **c, d**. Assigning the sleep or wakefulness label randomly results in random performance of the classifier for the same cell subtype.

To further probe the differences between closely related KC and glial subtypes, we applied a complementary, independent approach to differential expression analysis. We asked whether a tree-based classifier could learn the transcriptome makeup of one cell subtype during the sleep and wakefulness states and subsequently, how accurately this model would perform in classifying cells of another closely related subtype into either of these states (Fig. 4c). This approach was applied to the KC and glial cell subtypes separately. To ensure that the classifier would discriminate between sleep and wakefulness states rather than cell identity, we excluded marker genes between either KC or glial subtypes up to the point that the subtypes merged into one another in the two-dimensional uniform manifold approximation and projection (UMAP) space (Extended Data Fig. 6a,b). The accuracy of the classifier is illustrated in a confusion matrix, where the color corresponds to the probability to accurately assign a sleep or wakefulness label per cell type. We found that the classifier performed better for the cell subtype it was trained on, than on other related ones (Fig. 4c). To ensure that the classifier’s improved performance was not driven by overfitting to the cell type (that is, learning cell identity features), we randomly shuffled the sleep and wakefulness labeling of cells within their subtype identity. Then, the classifier would not distinguish between the sleep and wakefulness states even in the same subtype, suggesting that the classifier truly learned sleep/wakefulness, rather than cell identity features (Extended Data Fig. 6c,d). Taken together, both the differential expression analysis and classification approach suggest that depending on their cell identity, different cell populations have different sleep/wakefulness correlates.

**Extended Data Fig. 7:**
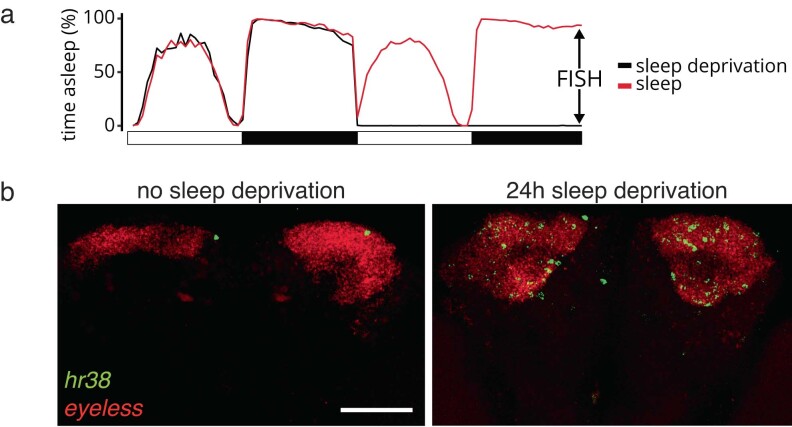
Validation of candidate sleep/wake correlate HR38 in KC. **a**. Sleep and sleep deprivation behaviour traces of flies used for candidate gene validation (sleep: n = 236, SD: n = 213). Arrows indicate time of sampe collection. **b**. Representative images of validating candidate gene *HR38* with KC marker gene *eyeless* by fluorescent *in situ* hybridisation. Results were consistent across brains (sleep: n = 4, SD: n = 5). Scale bar, 50 µm. Source data

Next, we asked what kind of genes are altered between sleep and wakefulness in glia and KCs. To gain confidence in the candidate gene list, we first trained another classifier to detect cell identity features between glial and KC subtypes. Then, we subtracted those cell identity features from those used by the sleep–wakefulness classifier (Fig. 4d,g). To reduce the false positive rate further, we considered genes as significantly deregulated only if they remained after the filtering step above and if they were identified as significant in the differential expression analysis, leaving 30 and 39 sleep/wakefulness-correlated transcripts in glia and KCs, respectively (Fig. 4d,g). Among the filtered 39 sleep/wakefulness correlates of KCs, we found *Hr38*, an activity-dependent gene in insects^33^. To validate its increased expression during SD and wakefulness compared to sleep in KCs specifically, we performed fluorescence in situ hybridization (FISH) during sleep and SD. In accordance with our single-cell data, *Hr38* mRNA in KCs increased expression after SD (Extended Data Fig. 7). The sleep/wakefulness correlates in glia included metabolism-related genes (*CG9360*, *Cyp28a5*, *GstE12*, *GstE1*, *mdh1*, *Ssadh* and *Ugt86Dd*), genes involved in protein synthesis and homeostasis (*Rpl41*, *Cct2*, *CG34362*, *CG6770* and *sm*), and genes regulating the core circadian clock (*CG31324* and *vri*) (Extended Data Fig. 5c and Supplementary Table 4). In contrast, sleep/wakefulness correlates in KCs included many genes involved in axon and synapse development and function: *Ten-a*, *rho*, *pyd*, *hig*, *Gfrl*, *CG42674*, *Hasp* and *beat-IIIb* (Extended Data Fig. 5c and Supplementary Table 4).

### Template matching captures cell types involved in process S

**Fig. 5:**
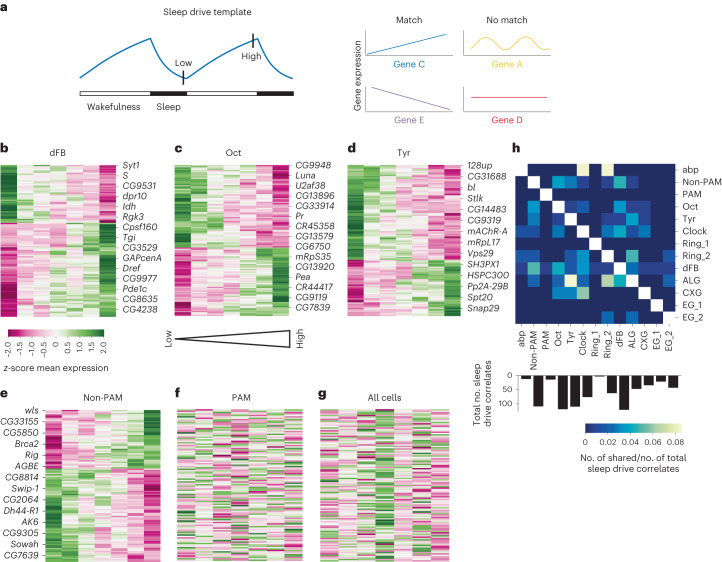
Analysis of molecular correlates of sleep drive. **a**, Illustration of the sleep drive template based on Borbély’s two-process model^1^. Points of low and high sleep pressure are indicated. Examples of genes whose expression would match (blue and purple) or not match (red and yellow) the template are given. **b**–**g**, Cluster map of all significant sleep drive correlates of dFB (**b**), Oct (**c**), Tyr (**d**) and non-PAM DANs (**e**). The gene expression of sleep drive correlates from non-PAM DANs did not correlate with the sleep drive template in PAM DANs (**f**) or all cells combined (pseudobulk) (**g**). A subset of significantly correlating sleep drive genes is labeled. The statistical test used was Pearson correlation. *P* values were adjusted for multiple tests using the Benjamini–Hochberg method. **h**, Heatmap of intersecting sleep drive correlates between all annotated cell types with at least one shared gene relative to the total number of sleep drive correlates according to cluster.

While the sleep/wakefulness correlates detect how the transcriptome changes in a binary manner, we next assessed whether it also changed gradually with the level of sleep pressure (sleep drive correlates). We sampled flies at multiple sleep and SD states, which can be ordered according to their level of sleep drive, from 20 h of GBX-induced sleep to 20 h of SD (Fig. 5a). To detect transcripts whose expression correlated with the gradual increase of sleep drive, we adopted a feature selection method^34^. Like the JTK algorithm for the identification of cyclers, which calculates the correlations between measured gene expression and assumed sine waves, this method also asks whether gene expression changes according to a given pattern. More specifically, we tested whether the expression profile for each measured transcript across sleep drive states, significantly correlates with a predefined template. The template consisted of values from 0 to 1, where 0 corresponds to the condition of lowest sleep pressure and 1 to the highest. Another five conditions were assigned to continuous values between 0 and 1 according to the respective level of sleep pressure the animal experienced (Fig. 5a and Methods).

**Extended Data Fig. 8:**
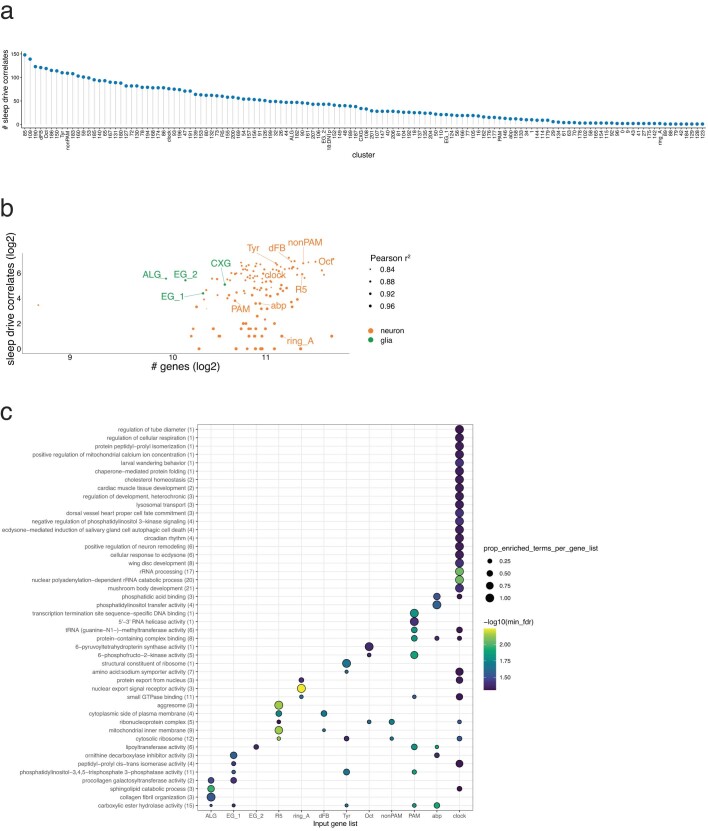
Sleep drive correlates across all clusters. **a**. Number of sleep drive correlates across all clusters with at least one correlate. **b**. Correlation between number of genes and number of sleep drive correlates for neuronal and glial cell types. **c**. Gene ontology parent terms enriched across annotated clusters. Number of child terms per parent term in brackets. Size of dot indicates fraction of child terms associated with the parent term.

We found that 65.1% of all clusters and 68.4% of annotated clusters had at least one sleep drive correlate (Extended Data Fig. 8a and Supplementary Table 5). Interestingly, the four annotated clusters with the highest amount of sleep drive correlates were cell populations associated with sleep homeostasis, spearheaded with 121 correlates by dFB neurons (Fig. 5b and Extended Data Fig. 8a,b), which we previously annotated (Extended Data Fig. 3). Similarly, wakefulness-promoting Oct, Tyr and non-PAM DAN neurons each had more than 100 sleep drive correlates (Fig. 5c–e and Extended Data Fig. 8a,b). Plotting the gene expression of sleep drive correlates from low to high sleep pressure conditions showed clear correlating patterns for dFB, Oct, Tyr and non-PAM DAN neurons (Fig. 5c–e). In contrast, the related dopaminergic subtype of PAM neurons only had 14 correlates (Extended Data Fig. 8a,b). Furthermore, the expression of non-PAM DAN sleep drive correlates did not correlate with sleep pressure in PAM DAN neurons nor in all cells combined (pseudobulk) (Fig. 5f-g). Thus, in this study we demonstrated that the template-matching method was sufficiently sensitive and specific to capture sleep drive correlates of previously identified sleep circuits.

Analogous to asking whether cyclers and sleep/wakefulness correlates differed between cell populations, we next assessed whether molecular correlates of sleep drive varied depending on cell identity. We found that the specificity of sleep drive correlates to a cell type was even more pronounced compared to that of the sleep/wakefulness correlates and cyclers. The highest overlap across all clusters was merely 8% between abp-KC and clock or ring_2 and between astrocytes (ALGs) and Tyr neurons (Fig. 5h). These data suggested that different cells responded to process S in unique ways.

Similarly, GO analysis revealed little overlap of sleep drive correlates between cell types (Extended Data Fig. 8c). In dFB neurons, we found many sleep drive correlates that were involved in synaptic formation and function (*unc-104*, *scramb1*, *dpr10*, *dpr19*, *syt1*, *sm*, *brp* and *CG8386*). This is consistent with previous evidence linking neuronal activity of dFB neurons to levels of sleep pressure^21^. Our data also showed that the expression level of several mitochondria-related genes also correlated with sleep drive, including *mRpL27*, *mRpL50*, *Sod2*, *CG32113* and *Idh*. This is in agreement with the notion that mitochondrial function in the dFB is critical for sleep homeostasis^35^.

### R5 neurons have a high number of sleep drive correlates

**Fig. 6:**
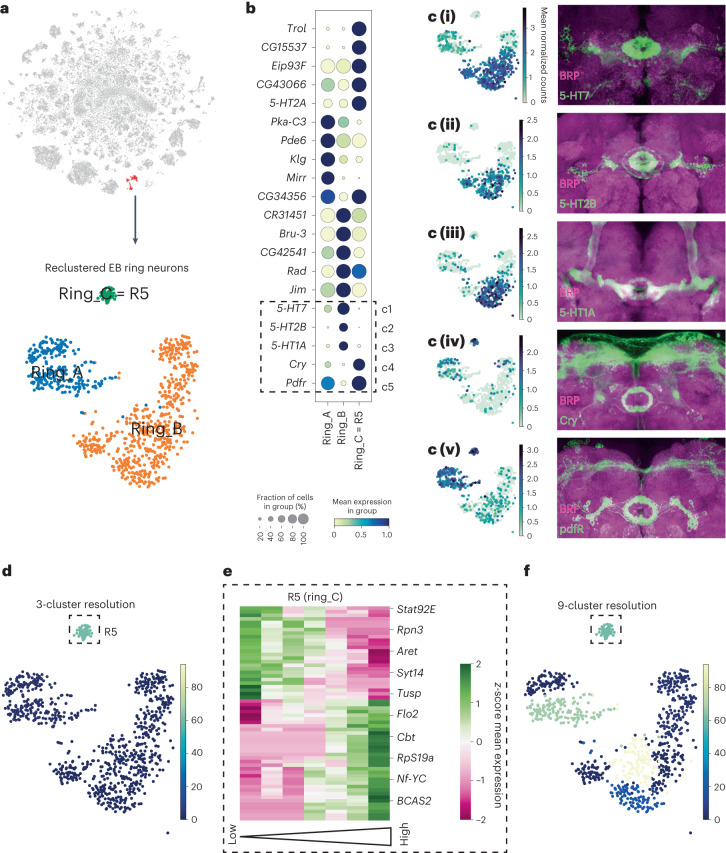
Identification of EB R5 neurons and sleep drive correlates across EB ring neuron subtypes. **a**, Top, *t*-SNE plot of two EB ring neuron subclusters. Bottom, reclustering of EB ring neurons into three subclusters, that is, ring_A, ring_B and ring_C. **b**, Top 20 DEGs between the three subclusters. **c** (i)–(v), Expression pattern and chemical tag staining of T2A-Gal4 driver lines of the genes boxed in **b**. Their morphology revealed that neurons in ring_C are EB R5 neurons. **d**, *t*-SNE displaying the number of sleep drive correlates at three-cluster resolution. **e**, Cluster map displaying the expression of sleep drive correlates for R5 neurons. A subset of significantly correlated sleep drive genes is labeled. **f**, *t*-SNE displaying the number of sleep drive correlates at a nine-cluster resolution. Source data

We found that one subcluster of EB ring neurons (ring_2) had a substantial number of sleep drive correlates, while the other (ring_1) showed only a few (Fig. 5h). Therefore, we asked whether the previously identified sleep drive-regulating R5 neurons are part of the ring_2 subcluster. R5 neurons contain only approximately 32 cells in an adult fly brain^19^. To identify them, we first selected all EB ring neurons that were annotated based on previously used marker genes^15^ and repeated dimensionality reduction exclusively on the EB ring neuron cluster to specifically analyze its subclusters. This reclustering resulted in at least three separate subclusters expressing unique transcriptomic signatures (Fig. 6a). To identify R5 neurons among the three clusters, we focused on a subset of marker genes (Fig. 6b), whose expression pattern is known from T2A-Gal4 knock-in driver lines^36,37^. By crossing relevant driver lines to chemically tagged effectors, we visualized the expression pattern of multiple marker genes in the EB ring neuron subclusters (Fig. 6c). EB ring neuron subtypes can be distinguished by their projection patterns into the ring-shaped EB structure^38^. This allowed us to map the subtype identities to some of our single-cell EB ring neuron subclusters. Ring_C differentially expressed *cry* and *pdfR* (Fig. 6b), two genes that are expressed specifically in neurons projecting into the R5 ring (Fig. 6c). Therefore, we identified cluster ring_C as R5. Remarkably, we found a high number of sleep drive-correlated genes specifically in R5 neurons, while few to no genes were identified in the other two subclusters (Fig. 6d,e and Extended Data Fig. 8a,b). Many of these transcripts were mitochondrial genes, including mt:ATPase6 and Pink1, as confirmed by GO analysis (Extended Data Fig. 8c and Supplementary Table 5). This finding suggested that mitochondrial function in R5 neurons is important for the role of these neurons in sleep homeostasis. Interestingly, we found that a gene encoding a potassium channel *ether-à-go-go* (*eag*) correlated negatively with sleep drive in R5 neurons. Potassium channels, including Eag, reduce neuronal excitability^39^. This is consistent with the finding that the neuronal activity of R5 increases with the levels of sleep drive^19^.

Anatomic analyses showed that there were 11 subclusters of EB ring neurons^40^. Therefore, we asked whether our two bigger EB ring neuron clusters still contained multiple subpopulations and reclustered them into another eight subclusters. Coloring each cell by its assigned run showed that no clusters were dominated by one run, thereby confirming that our EB ring subclusters were indeed driven by different cell subtype identities rather than by technical batch effects (Extended Data Fig. 2c,d). Matching the sleep drive template to these subclusters revealed that two of them had a high number of correlating genes in addition to R5 (Fig. 6f). This is in line with studies that identified additional ring neuron subtypes apart from R5 that regulate sleep amount and sleep fragmentation^20,41^. Importantly, the remaining six subclusters had no or few genes correlating with sleep drive (Fig. 6e), demonstrating the specificity of the template-matching method. This reclustering analysis also highlighted the importance of examining homogeneous populations.

### Clock neuron subtypes differ in sleep drive correlate numbers

**Fig. 7:**
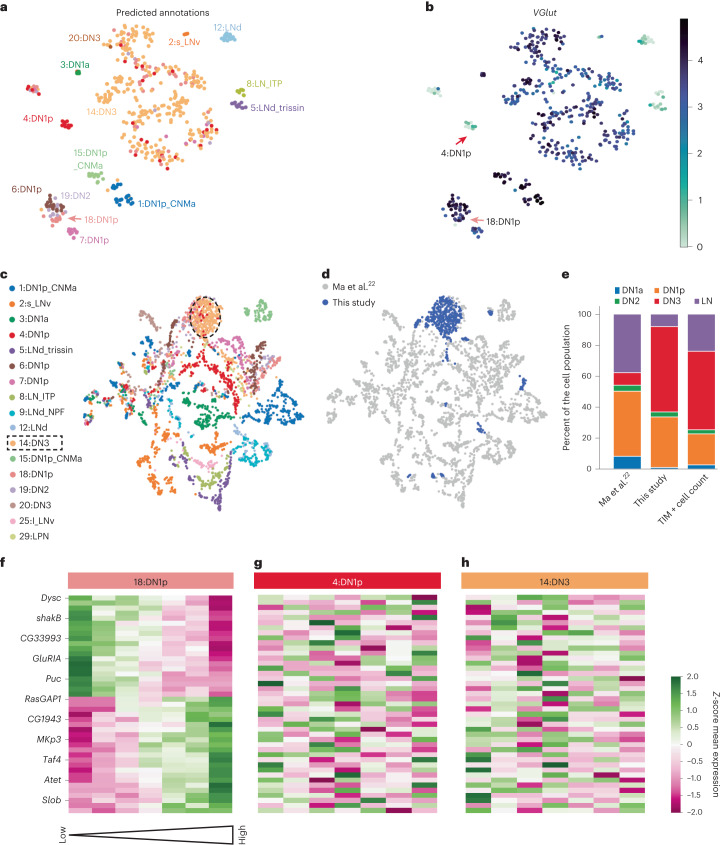
Analysis of sleep drive correlates in clock neuron subtypes. **a**, Predicted annotations of clock neuron subtypes on our data based on training the scANVI model on data from Ma et al.^22^. Cluster annotation names were transferred from Ma et al.^22^. **b**, *VGlut* expression mapped onto our data; DN1p subtype clusters 18 (green) and 4 (red) are indicated. **c**, Plotting of our 494 clock neurons together with clock scRNA-seq atlas of Ma et al.^22^ split into 17 high-confidence annotated clusters. **d**, Our cells in blue highlighted in the UMAP space with data from Ma et al.^22^. **e**, Proportion of cells according to clock subtype for data from Ma et al.^22^, data from this study and published counts of cells from neuroanatomical studies. **f**–**h**, Cluster map of all significant sleep drive correlates of 18:DN1p (**f**). Gene expression of the significant genes for 18:DN1p did not correlate with sleep drive in another DN1p subtype (**g**) or 14:DN3 (**h**).

Clock neurons are a heterogenous population consisting of four dorsal neuron subtypes (DN1a, DN1p, DN2 and DN3) and three lateral neuron (LN) subtypes (LNv, LNd and LPN)^42^. Previous anatomical analyses and single-cell transcriptomic data also suggested that many of these clock neuron subtypes, especially DN1p, can be further divided into smaller clusters^22,43^. Importantly, some clock neuron subtypes are involved in sleep regulation, while others are not^44–47^. We aimed to investigate if we could detect this discrepancy in our data. To this end, we first subclustered our 494 clock neurons using an approach similar to that used with the EB ring neurons. We found clear subclusters emerging in the dimensionality reduction (Fig. 7a and Extended Data Fig. 2e,f). Like subclusters of the EB ring neurons, clock neuron subclusters were driven by different cell identities rather than by technical batch effects (Extended Data Fig. 2e,f).

**Extended Data Fig. 9:**
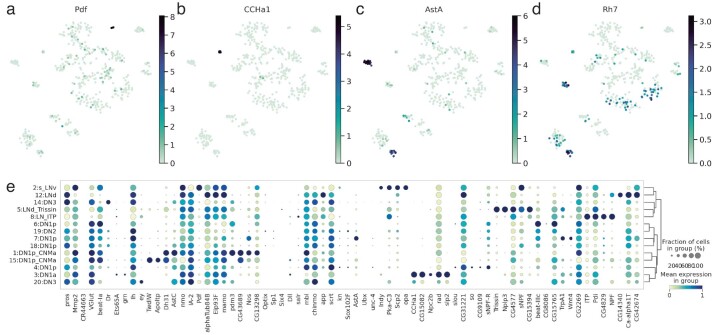
Marker genes of clock neuron subtypes. **a-d**. Marker gene expression mapped onto our data, confirming model predictions. **e**. Top 5 marker genes per cluster identified in Ma et al.^22^ and their gene expression in our data.

To clearly annotate our clock neuron subclusters, we trained a semi-supervised model (single-cell annotation using variational inference (scANVI))^48^ on the 2,615 single-cell transcriptome profiles of the published clock neurons by Ma et al.^22^. The model was then used to predict the clock subcluster identities of our 494 clock neurons (Fig. 7a). Ma et al.^22^ annotated 17 clock neuron subclusters with high confidence. We found 14 of the 17 high-confidence subclusters in our data (Fig. 7a). We confirmed the expression of some cluster-specific genes (*Pdf* for s_LNv, *CCHa1* for DN1a, *AstA* and *Rh7* for DN1p, and *vGlut* for all clusters except for all LN clusters and 4:DN1p) (Fig. 7b and Extended Data Fig. 9a,d). We also tested the expression of the top five marker genes identified by Ma et al.^22^ across all 14 annotated clusters. Like their findings, we observed that within the same subtype of DN1p neurons, six subclusters with distinct gene expression signatures emerged (Extended Data Fig. 9e). Furthermore, most of our subclusters that had emerged using dimensionality reduction were clearly mapped to individual clock neuron subtypes, particularly the LNs and DN1p subclusters (Fig. 7a). These findings indicate that we successfully annotated our clock neuron subclusters.

However, the prediction was less successful when separating the 19:DN2 subcluster because it clustered together with the 6:DN1p and 18:DN1p subtypes (Fig. 7a). Furthermore, the predicted 14:DN3 cluster divided into multiple clusters (Fig. 7a), suggesting the existence of multiple DN3 subtypes that the study by Ma et al.^22^ may have missed. We propose that the larger number of DN3 neurons in our dataset (Fig. 7c,d) could be due to the difference in methodologies between the studies. Ma et al.^22^ FAC-sorted clock neurons by using the Clk856-Gal4 driver, which does not label most DN3 neurons. In contrast, our data were generated in an unbiased manner, that is, not by sorting a driver line. In accordance, the proportion of clock subtypes we yielded matches the number of cells that others have previously counted based on immunostaining of TIMELESS protein^43,44^ (Fig. 7e). In contrast, but as expected, the data sorted from the Clk856-Gal4 driver consisted of only a small proportion of DN3 cells. Interestingly, compared to the anatomical cell counts, we lacked LNs (Fig. 7e). This is probably because these cells may have been removed together with the optic lobe when we dissected the central brains, as LNs are located close to the border between the central brain and optic lobes.

Like the homogenous R5 subcluster, we next asked whether we could identify sleep drive correlates in the more homogenous, annotated clock neuron subclusters. However, while the template-matching method works well on homogenous populations, it still requires a minimum number of cells such that all template conditions are covered. This limitation resulted in the template matching to run only in three annotated clock subclusters, that is, 18:DN1p, 4:DN1p and 14:DN3. Interestingly, we identified significant sleep drive correlates only in the glutamatergic-positive cluster 18:DN1p, not in the glutamatergic-negative 4:DN1p subtype nor in 14:DN3 neurons (Fig. 7f–h). This is consistent with findings that glutamatergic DN1p neurons are involved in sleep/wakefulness regulation^44,45^. Among the sleep drive correlates of the 18:DN1p cluster are two genes that regulate the function of a potassium channel: *slowpoke-binding protein* (*slob*) and *dyschronic* (*dysc*) (Fig. 7f). This is consistent with the finding that the potassium channel (slowpoke) is important in glutamatergic DN1p neurons to regulate sleep quality^49^.

### Homeostatic and circadian processes converge in glia

**Fig. 8:**
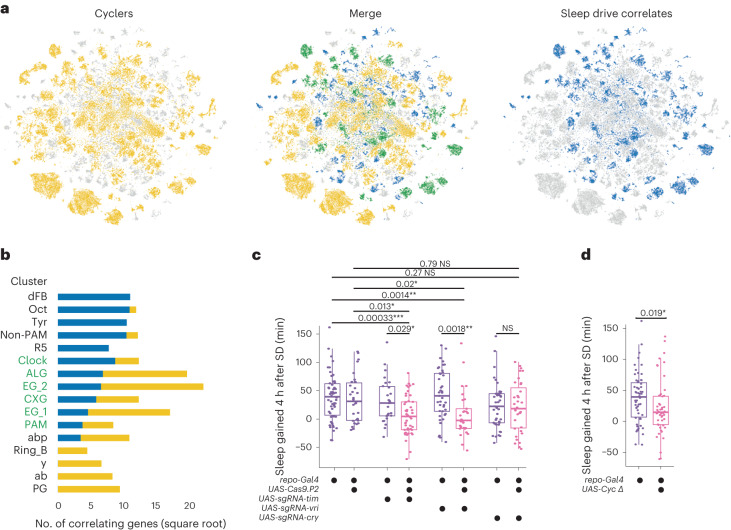
Homeostatic and circadian processes converge on glial cells. **a**, Clusters assigned to cyclers (yellow, left) or sleep drive correlates (blue, right) visualized in the *t*-SNE. Middle, The *t*-SNE shows the merge of left and right *t*-SNE, highlighting the clusters assigned to both groups in green. **b**, Number of correlating genes with circadian or sleep drive template across all annotated clusters that have correlates for either process. The color indicates their assigned group (yellow, cyclers; blue, sleep drive correlates; green, both). **c**, Sleep amount 4 h after SD compared to the baseline sleep of the same fly in the same ZT time period before SD in flies expressing conditional knockout of *vri* (*n* = 30), *tim* (*n* = 47) and *cry* (*n* = 35) in pink and control flies (*repo-Gal4>iso31* (*n* = 58); *repo-Gal4*>*UAS-Cas9.P2* (*n* = 30); *repo-Gal4*>*UAS-sgRNA-vri* (*n* = 41); *repo-Gal4*>*UAS-sgRNA-tim* (*n* = 26); and *repo-Gal4*>*UAS-sgRNA-cry* (*n* = 41)) in purple. **d**, Sleep amount 4 h after SD compared to baseline sleep of the same fly in the same ZT time period before SD of flies expressing a dominant negative form of *Cyc* (*n* = 48) in pink and control flies (*repo-Gal4>iso31* (*n* = 58)) in purple. **c**,**d**, The statistical method used was a Wilcoxon rank-sum test, with Bonferroni-corrected *P* value adjustment for multiple-comparisons testing. The boxplots indicate the minimum, median, maximum, and first and third quartiles. The error bars represent the first (third) quartile ± 1.5 times the interquartile range. Adjusted *P < 0.05, **P < 0.01, ***P < 0.001; NS, not significant. Source data

Previously, it was shown in flies that homeostatic and circadian processes interact indirectly by neuronal circuits connecting clock neurons with sleep homeostat circuits (EB ring neurons and dFB neurons)^44–46,50,51^. We asked whether there is a more direct interaction of the two processes in the same cell and whether one process affects a cell type more than the other one. To this end, we compared the number of sleep drive correlates with the number of cyclers by cell population and assigned each cell type to either process or to both simultaneously (Methods). Surprisingly, in most cases, we found that a given cell type was more affected by only one process (Fig. 8a,b). For example, dFB, Oct/Tyr, non-PAM DANs and R5 neurons had many sleep drive correlates but few circadian cyclers. On the other hand, cell types with many circadian correlates, for example, y-KCs, ab-KCs and PGs, had no sleep drive correlates. Remarkably, two subtypes of EB ring neurons, that is, R5 and ring_B were affected by either process in opposing ways, in accordance with previous findings, showing that R2/R4m neurons (probably part of ring_B) received circadian timing information from clock neurons^51,52^, while R5 neurons themselves encoded the sleep homeostat^19^. This suggested that the effect of either circadian or homeostatic process differs depending on the cell type and can vary even for closely related cell types. KC subtypes, except for abp-KCs, followed a pattern of high number of circadian cyclers, but few or no sleep drive correlates. Intriguingly, both the number of cyclers and sleep drive correlates were high in all glia with the exception of PGs, as opposed to few neuronal clusters with such high numbers. This demonstrated that a simultaneous convergence of both circadian and homeostatic processes takes place in glial cells, as their transcriptome is affected by both.

Interestingly, we identified genes that regulate the core molecular clock, *vri* and *CG31324*, as sleep/wakefulness correlates in glia. Similarly, the expression levels of *E23*, a regulator of the circadian rhythm, correlated with the sleep drive in glia specifically. Thus, process S may directly influence the core clock machinery in glial cells. Next, we asked whether the disruption of the circadian clock, specifically in glia, would result in impairment of the sleep homeostat. We expressed a dominant negative form of *Cyc*^53^ or conditionally knocked out *tim*^54^, *vri* or *cry*^55^ specifically in glial cells, while leaving all neurons, including clock neurons, unaffected. To assess sleep homeostasis in these animals, we sleep-deprived flies for 12 h during the night and measured rebound sleep, a hallmark of sleep homeostasis, the following morning. Flies with a disrupted glial clock showed significantly reduced rebound sleep after SD compared to control flies (Fig. 8c,d). Interestingly, while disrupting expression of *tim*, *vri* and *Cyc* resulted in reduced sleep rebound, *cry* knockout did not. This is in accordance with *cry* participating in neither of the two core transcriptional–translational feedback loops in *Drosophila*. These data indicate that the glial clock is required for normal sleep homeostasis and suggest that processes S and C directly influence each other in glial cells to determine sleep–wake cycles.

### Interface for browsing gene expression correlates

**Extended Data Fig. 10:**
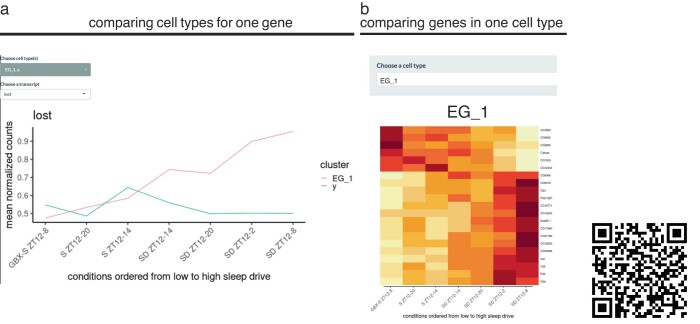
Webtool to browse gene expression across sleep conditions and ZT times. **a**. Comparing expression of given gene across different cell types. **b**. Comparing expression of significant correlates in given cell type. Examples shown are based on sleep drive correlates. Cyclers and sleep vs wake correlates are available in the webtool, see QR code.

Our correlational analyses (cyclers, DEGs between sleep and wakefulness, and correlates of sleep drive) across all genes and clusters are accessible to explore visually within a user-friendly interface (https://www.flysleeplab.com/scsleepbrain) as a complement to Supplementary Tables 3–5. The expression of a gene of interest can be compared across cell types (Extended Data Fig. 10a) or in relation to other genes of the same cell type (Extended Data Fig. 10b).

## Discussion

The present study is the first to profile gene expression across different sleep and wakefulness states, degrees of sleep pressure and diurnal time points in an unbiased manner across all cell populations of an entire central brain. We show that sleep/wakefulness and sleep pressure as well as circadian cycling correlates can be found at the transcriptional level across the 214 clusters, including 20 annotated clusters. Few correlates overlap between clusters, which suggests that distinct cell populations are affected differently by sleep/wakefulness, sleep drive and circadian time. We further show that many correlates are only visible in homogenous populations and not in a pseudobulk sample, probably because correlates are largely unique to a specific (sub)cluster. Particularly for sleep drive correlates, the higher the granularity of a cluster, the more correlates are captured, again highlighting the importance of cluster homogeneity in such analyses. Further support for the notion that molecular correlates are only visible at single-cell resolution stems from the observation that the results of previous bulk transcriptomic studies^22^, which have profiled transcriptome changes across the sleep–wake cycle in *Drosophila*, neither overlap with our results nor each other’s results. Similarly, our pseudobulk sleep/wakefulness correlates only overlap by a maximum of 2% compared to their results (Supplementary Fig. 1).

While providing an unbiased view of all cell types in the fly brain, droplet-based scRNA-seq methods as used in this study are known to have less sequencing depth and power to detect rare cell populations compared to well-based methods^55^. Well-based methods specifically target cell types by prepurifying them with a driver line. Despite the limitations of droplet-based methods, we successfully subclustered and identified sparse neuronal subtypes in the brain from our dataset, including dFB, R5 and different clock neuron subclusters, such as sLNv neurons, which contain only eight cells per brain. Clock neuron subclusters and dFB neurons were annotated by making use of publicly available datasets generated with well-based technologies. In the future, an approach that combines both types of isolation methods will be important to identify additional rare subpopulations in our dataset.

We found that the core clock machinery exists only in clock neurons and glial cells, but not in other neurons in the fly brain. This is consistent with a previous report of the absent expression of core clock genes in *Drosophila* DANs^56^ and KCs^57^. At the same time, most of the neurons express cyclers. These cyclers in non-clock neurons are probably driven by cells containing the molecular clock, including clock neurons and glial cells. Glial cells may be a more suitable candidate for this role, considering their large numbers and coverage across the entire brain, in contrast to approximately 150 clock neurons, whose processes cover only a small proportion of the brain. Supporting this idea, the glial clock in mice has been shown to drive the circadian clock gene expression in the SCN and is sufficient to initiate and sustain circadian locomotor rhythms^58^. Similarly, in flies, perturbing glial release results in loss of circadian rhythmic morphological plasticity of pacemaker small ventral LNs^18,58^. Thus, the circadian cyclers in many non-clock neurons in flies may be driven by the molecular clock in nearby glial cells.

By using single-cell transcriptomics and template matching, we detected gene expression changes associated with different sleep drive levels in real time, while sleep pressure accumulates for all cell types separately. Surprisingly, many clusters showed a high number of transcriptional sleep drive correlates, including R5 and dFB neurons, which are directly involved in sleep homeostasis, and the wakefulness-promoting Tyr, Oct and non-PAM DANs, as well as a subset of glutamatergic DN1p clock neurons that interact with the sleep homeostat. Furthermore, many sleep drive correlates are captured in glial cells, several subtypes of which have previously been shown to regulate sleep/wakefulness or sleep homeostasis in *Drosophila*^18,59^. Our findings of sleep drive correlates illustrate the high specificity of the method to identify relevant sleep homeostasis regulating circuits, even when they are small (sub)populations. Therefore, other yet unannotated clusters with a high number of sleep drive correlates may also be involved in the homeostatic regulation of sleep; it will be interesting to determine their identity to ultimately examine their sleep-regulating function.

Sleep homeostasis and circadian rhythms are two distinct behavioral processes and probably affect different cells in the brain. Accordingly, we found that most cells in the fly brain have either a high number of sleep drive correlates but a low number of circadian cyclers, or the reverse. However, although the homeostatic and circadian processes are known to function independently, increasing evidence suggests a cross talk between these two processes^60,61^. In flies, recent studies suggested a model in which the sleep homeostat and the circadian clock interact indirectly through neuronal circuit connections. Several circuits convey the circadian time information from clock neurons to the sleep homeostat centers EB and dFB^44,45,52^. Reciprocally, hugin^+^ neurons act downstream of dFB neurons and modulate clock neurons^50^. However, our data argue for a parallel regulatory mechanism, in which sleep homeostatic and circadian processes interact directly in glial cells to regulate the sleep–wake cycle. In addition, this glial mechanism and the previously identified neuronal mechanisms may also reciprocally interact with one another to integrate processes C and S in sleep–wake regulation. In this glial mechanism, we propose that the sleep–wake cycle affects the regulators of core clock genes in glia, and that the molecular clock in glia is required for sleep homeostasis. How do these two processes interact in glial cells? We and others previously demonstrated that glial Ca^2+^ signaling encodes the level of sleep needed^18,59^. In addition, Ca^2+^ signaling has an important role in regulating the oscillation of core clock genes, with many Ca^2+^ channels and transporters rhythmically expressed in mammalian clock neurons^62^. Thus, the reciprocal interaction between Ca^2+^ signaling and the molecular clock in glia may be the molecular substrate of the interaction of homeostatic and circadian processes to ultimately instruct downstream neurons and appropriate behavior.

## Methods

### Animals

The following *Drosophila* strains were obtained from the Bloomington *Drosophila* Stock Center: DGRP 88, 287, 303, 313, 359, 379, 441, 646, 892 and 908; *5-HT1A-Gal4* (strain no. 84588); *Pdfr-Gal4* (strain no. 84684); *cry-Gal4* (strain no. 24514); *UAS-Cyc Δ* (strain no. 36317); *iso31* (strain no. 5905); *repo-Gal4* (strain no. 7415); and *UAS-sgRNA-tim3x* (strain no. 90768). *UAS-3x-vri-g* and *UAS-3x-cry-g* were a gift from M. Rosbash^55^. *5-HT2B-T2A-Gal4* and *5-HT7-T2A-Gal4* were a gift from S. Kondo^37^.

### Sleep behavior

After entraining male flies for 3 days, 4–8-day-old single flies were loaded during the active period between ZT0 and ZT2; sleep was recorded in 12:12 light–dark conditions in a constant environment of 55–65% humidity and 22 °C. Locomotion was tracked in a high-resolution video-based Raspberry Pi-enabled device (ethoscope)^63^. Targeted mechanical SD was delivered in a feedback loop triggered by 10 s of quiescence of a fly by the rotation of the respective tube. Sleep analysis was performed with adaptations to the rethomics pipeline in R.

For sleep profiling of the DGRP lines, 36 DGRP lines were selected based on sleep architecture metrics previously reported in 168 DGRP lines^12^. For the 36 lines, we thoroughly compared sleep amount, latency, fragmentation and depth. We selected those lines with sleep patterns similar within and between the lines. PCA and analysis of pDoze and pWake^13^ were performed in R. The plots in Extended Data Fig. 1 were created in Python and R. Flies that slept less than 50% of the average amount of their genotype were excluded (4.5%, 37 of 812 flies).

For the scRNA-seq experiments, around 200 flies were loaded belonging to different combinations of the behavioral conditions linked to one or two of the ten DGRP lines in each of seven runs. Flies belonged to 2–4 conditions and 5–9 DGRP lines. The link between condition and DGRP line was shuffled in every run. Flies were preselected based on the recorded baseline sleep and wakefulness behavior on the day before dissection. Immediately before dissection, a final selection of 40 flies was decided based on the previous day’s preselection and the sleep or wakefulness behavior up to the point of dissection in real time. Dissection, dissociation and 10x processing followed immediately after selection.

To validate candidate sleep/wakefulness correlates, flies (DGRP 379/iso31) were collected at ZT0 either after 12 h of normal sleep or 12 h of targeted SD in ethoscope devices. Fly selection and monitoring of their sleep were performed as described above. Brain dissection and whole-mount FISH (see details below) followed immediately after fly collection; both conditions were processed in parallel. For each candidate gene, the brains of at least four 5–7-day-old male flies per condition were processed.

Sleep rebound was assessed after 12 h of targeted SD between ZT12 and ZT24 and one or two prior baseline days in 4–7-day-old mated females in constant darkness conditions. Each genotype was tested in a least four independent experiments. Genotypes were distributed randomly across ethoscopes, ensuring that each genotype was never exclusively tested in a single ethoscope, thereby avoiding potential technical effects. Gained (or recovery) sleep was calculated as the difference between the minutes asleep during the first 4 h after SD and the minutes asleep during the same 4-h circadian time period (ZT0–ZT4) from the two (average) or one baseline days. Genotypes were compared with each other by performing a nonparametric Wilcoxon rank-sum test. Multiple-comparisons testing was Bonferroni-corrected. We excluded animals that slept more than 0.25% of the time during the SD period and flies that slept less than 30% of the average sleep amount of their genotype during the baseline days.

### Brain dissociation into single cells

In each of seven replicates, around 40 central brains were dissected in ice-cold Schneider’s medium with 30 mM of the transcription inhibitor actinomycin D (catalog no. A1410, Sigma-Aldrich). The number of brains per run was chosen based on previous work^14^; this number ensured that the main cell types would be represented. Within 45 min after starting the dissections, the brains were dissociated with an enzyme mixture of dispase (3 mg ml^−1^), collagenase (100 mg ml^−1^) and trypsin-EDTA (0.05%) in a thermoshaker at 25 °C for 15 min. The dissociation was reinforced by pipetting the solution at least four times during the incubation. After washing with PBS with 15 mM actinomycin D the cell pellet was resuspended in 100–200 ml PBS with 0.04% BSA and filtered with a 10-mM pluriStrainer (catalog no. 435001050, ImTec Diagnostics).

### 10x Genomics

Library preparations for the scRNA-seq was performed using the 10x Genomics Chromium Single Cell 3′ Kit v.3.1 NextGEM chemistry (10x Genomics). The cell count and viability of the samples were assessed using a LUNA Dual Florescence Cell Counter (Logos Biosystems). For each sample, a targeted cell recovery of 10,000 cells was aimed for. The dissociated cells from a fly brain are typically quite small (averaging between 1 and 4 µm); this affects counting accuracy. To get consistent counting accuracy, the average of cell counts from three independent measurements were considered for loading the fly cells to the 10x controller. After the cell count and quality check, samples were immediately loaded onto the Chromium Controller. scRNA-seq libraries were prepared according to the manufacturer’s recommendations (Single Cell 3′ Kit v.3.1 user guide; CG000204 Rev D); at the different check points, library quality was assessed using a Qubit (Thermo Fisher Scientific) and Bioanalyzer (Agilent Technologies). For a targeted sequencing saturation of 50–60%, sequencing was performed at a depth of 30,000–60,000 reads per cell; single-cell libraries were sequenced either on Illumina’s NovaSeq 6000 platform or HiSeq 2500 platform using a paired-end sequencing workflow and with recommended 10x v.3.1 read parameters (28-8-0-91 cycles).

### 10x Genomics data preprocessing

The 10x data were mapped to the *Drosophila melanogaster* (BDGP6 assembly) genome using CellRanger v.3.1.0. SNP data for the DGRP lines was downloaded from http://dgrp2.gnets.ncsu.edu/. liftOver was used to convert these to dm6 coordinates. SNPs were then filtered to include only those present uniquely in one of the lines used. This VCF file was used to run demuxlet from the popscle package (https://github.com/statgen/popscle) with default parameters; tools from https://github.com/aertslab/popscle_helper_tools were used to speed up computation.

### Data processing

Scanpy v.1.4.4 was used to process the 10x libraries; samples were first loaded and cells were annotated with the genotypes determined by demuxlet. Demuxlet determined any doublets; ambiguous cells were removed and any cells assigned to a genotype not present in that experiment were also removed. Finally, scrublet was used to remove any remaining doublets using an expected doublet rate determined by the following formula based on the known doublet rate of the 10x Chromium device and the number of doublets expected to be remaining after demuxlet (within-genotype doublets): (0.008 × (n_cells/1000)) × (num_lines/(num_lines^2^)).

After doublet removal, cells with fewer than 200 genes were removed, as well as cells with more than 20–30% unique molecular identifiers assigned to mitochondrial genes (the thresholds can be found in Supplementary Table 7); cells belonging to the ZT2 Wake condition were also removed. All remaining cells were combined into a single sample for further processing. Cells were normalized to a total of 10,000 counts per cell and log-transformed; highly variable genes were identified using default parameters and the number of counts and percentage mitochondrial reads were regressed out. Finally, counts were scaled to unit variance with a zero mean with a maximum value of 10. A PCA was performed on the data. The pcacv workflow from the vsn-pipelines v.0.25.0 (10.5281/zenodo.3703108) was used to determine the number of principal components (58) to continue with further dimensionality reduction and clustering. Both UMAP and *t*-SNE dimensionality reductions were computed and clusters were determined using the Louvain algorithm at various resolutions (Supplementary Table 2). Final anndata (v.0.7.8) objects were converted to Loom files for visualization in SCope^14^.

### Multiplexing strategy

DGRP lines can be distinguished from one another by their unique SNPs. Their natural genetic variation from each other allowed us to determine the condition for each cell from the sequenced data^10^. This strategy has the added advantage of removing around 90% of droplets that enclose two cells instead of one from the dataset. This multiplexing strategy minimizes technical variation and batch effects, otherwise arising from separate complementary DNA library preparations.

### Integration of Smart-seq2 data of dFB neurons

To identify the cell cluster containing dFB neurons in our dataset, we made use of the publicly available transcriptome data of a FAC-sorted dFB subset genetically targeted with the R23E10-Gal4 driver. The raw data processed with a standard Smart-seq2 protocol was downloaded from the Gene Expression Omnibus (GEO) (accession no. GSE107451)^47^. Reads were cleaned using fastp v.0.20.0 and mapped to the BDGP6 *D. melanogaster* and quantified using STAR v.2.7.9a. A non-negative least squares regression model was used to match dFB Smart-seq2 cells to their corresponding cluster in our atlas (Extended Data Fig. 3); it was performed as described previously^48^, with the top ten marker genes (determined according to *z*-score) per cluster from resolution 0.8 being used as the gene subset.

### Clock cell subtype annotation with public dataset and scANVI

Raw data from Ma et al.^22^ were downloaded from GEO (accession no. GSE157504). Data were normalized to a total of 1 × 10^6^ reads per cell, log-normalized and the top 2,000 variable genes were identified using the ‘experiment’ annotation as a batch key. Annotations were obtained from the authors and a scANVI model^64^ was trained using these labels according to the scArches recommendations^65^. Briefly, an scVI model^66^ was first trained with ‘experiment’ as the batch key and raw counts as the input using the following parameters: use_layer_norm = ‘both’, use_batch_norm = ‘none’, encode_covariates = True, dropout_rate = 0.2, n_layers = 2, early_stopping = True, train_size = 0.8 and check_val_every_n_epoch = 1; a scANVI model was then initialized using the pretrained scVI weights, with the annotations as a label key and unlabeled categories set to ‘unknown’. Next, clock neurons were subset into their own adata object from the main dataset by selecting cluster 84 from a Louvain clustering with a resolution of 8.0. The trained scANVI model was then updated online; clock neurons and labels were predicted per cell. An integrated view of the two datasets was created by using the X_scANVI latent representation to calculate a neighborhood graph and subsequent UMAP.

### Downstream analyses

All analyses (differential expression analysis, sleep drive template matching and identification of cycling genes), except for the EBM classifier, were performed on all clusters with a high resolution of 8.0 of the Louvain clustering, except for those clusters annotated in a different resolution (Supplementary Table 2). Also, we excluded genes that displayed obvious expression differences between different runs for all analyses (Supplementary Table 6).

### Validation of circadian clock-related genes with SCENIC

Gene coexpression analysis was performed with single-cell regulatory network inference and clustering (SCENIC) using the scenic_multiruns workflow (pySCENIC v.0.11.2) from the vsn-pipelines in two steps (to decrease computational time). First, ten runs were performed with a list of all transcription factors; any transcription factors linked to a motif or track that were detected as a regulon by SCENIC were used to perform a second step of an additional 90 SCENIC runs only looking for these transcription factors. The area under the curve (AUC) value quantifies the presence of that motif in a cell type. We compared AUC values of the *Clk* regulon across all cell types.

### Identification of cycling genes

To identify cycling genes between ZT2, ZT8, ZT14 and ZT20, we used the JTK algorithm of the MetaCycle v.1.2.0 package (https://github.com/gangwug/MetaCycle)^66^. As MetaCycle takes a matrix of three replicates for each time point, we treated each cell in a cluster as a replicate and randomly assigned it to one of three pseudo-replicates for each ZT time point. The mean expression value for each combination of cluster and ZT time point replicate was calculated according to transcript. This matrix served as input for the JTK algorithm. The matrix was filtered to test only genes that had a maximum expression of 0.8 counts per million and an expression amplitude of at least 1.5-fold between time points, similar to a previous study^67^. We repeated the random allocation three times. Only clusters for which matrix generation was successful in all three technical replicates were considered for further analysis, leaving 183 of the 214 initial clusters. Matrix generation was successful in all annotated clusters. A gene was labeled as significantly cycling (Benjamini–Hochberg-corrected two-tailed *P* = 0.05) in a cluster only if it was detected as such in all three technical replicates. The threshold set to assign a cluster to the cyclers group (Fig. 8a) was 3.72, which is the mean of the square root of the number of cycling correlates for all 195 clusters.

### Differential expression analysis

The Wilcoxon rank-sum test was used to compare groups of cells for differential expression analysis; this was performed using the rank_genes_groups function from Scanpy, where n_genes is the number of genes in the dataset. Reference and test groups were set per differential expression analysis. Transcripts were considered DEGs if their Benjamini–Hochberg false discovery rate (FDR)-corrected *P* < 0.05. All cells except for KCs were divided into either cholinergic, GABAergic, glutamatergic or unknown based on their expression of *VAChT*, *Gad1* and *VGlut* above a value of 0.5, 0.4 and 0.5, respectively.

### Tree-based modeling of sleep and wakefulness states

We performed a binary classification task to learn to map the transcriptome of single cells to the behavioral labels ‘wakefulness’ or ‘sleep’. Specifically, we trained separate instances of the EBM, using identical training settings and hyperparameters, implemented in the interpret.glassbox.ebm.ExplainableBoostingClassifier class with default settings or as declared in scikit-learn v.1.0.2 and interpret v.0.2.7. Each instance was trained on a different cell subtype within a group of related cell types or ‘backgrounds’, specifically the glia or KC subtypes. Marker genes were excluded from the training to prevent classification based on cell identity as opposed to behavioral state. Marker genes were determined by performing differential expression analysis of either the glia or KC subtypes. This analysis resulted in excluding those genes with a log fold change greater than 3.5 (glia) and 2 (KCs) during training (Extended Data Fig. 6a,b). The trained models were then used to predict behavioral state on test datasets of each glial or KC subtype to compare classification performance on the same and different cell subtypes.

### Finding shared genes between DEA and EBM

The cutoff of the top features that resulted from the EBM classifier outlined above was decided by calculating the mean plus 2 s.d. across all features per subtype. Features falling above this threshold were regarded as significantly contributing to the classifier’s decision. The same thresholding was applied to the features used by the control classifier that predicted cell subtype identity. The resulting control features were then merged with the feature subsets generated in the first step. The remaining genes were then merged with all significant corrected *P* values of the DEGs from the differential expression analysis (see above) on either glia or KCs.

### Sleep drive template matching

Expression of all transcripts across different conditions were tested for a significant Pearson *r*^2^ correlation with a sleep drive template for each cluster separately. Sleep drive correlates below a Benjamini–Hochberg FDR-corrected *P* = 0.05 were considered significant. The template consisted of seven conditions ordered according to their respective amount of sleep or SD. A value between 0 and 1 was assigned to each condition according to that order with even intervals. Gene expression matrix generation across the seven conditions failed for 19 of the 214 clusters, although none of the annotated clusters. For the remaining 195 cell populations, for each transcript, we assessed whether there was a significant correlation between the sleep drive template and the expression value of each gene across the sleep and SD conditions (Fig. 5a). The threshold set to assign a cluster to the sleep drive correlate group (Fig. 8a) was 3.72, which is the mean of the square root of the number of sleep drive correlates for all 195 clusters.

### GO analysis

To identify the overarching GO terms of the resulting lists of significant correlates (cyclers, sleep/wakefulness or sleep drive correlates), we used the gprofiler2 R package v.0.2.1 with, as custom background, the detected genes of our sequencing data. Significant genes were ordered according to log fold change or Benjamini–Hochberg FDR-corrected *P* for sleep/wakefulness correlates, cyclers and sleep drive correlates, respectively. GO analysis was performed for each cluster separately for each of the three correlates. GO terms were subsequently grouped according to parent terms based on semantic similarity as calculated using the ‘Wang’ method^68^ in the mgoSim() function of the GOSemSim R package v.2.20.0 and the reduceSimMatrix() function in the rrvgo package v.1.6.0. Plotting was done with the go_reduce() and go_plot() functions of the rutils package v.0.99.2.

### Single-molecule FISH

Custom probes with hybridization chain reaction (HCR) technology were acquired from Molecular Instruments. The protocol was optimized based on the protocol for zebrafish larvae (from Molecular Instruments) and a whole-mount adult fly FISH protocol^68^. Briefly, brains were dissected in S2 medium, fixed in 2% paraformaldehyde (PFA) in PBS for 55 min, washed three times for 15 min in PBS with 0.5% Triton X-100, sequentially dehydrated and incubated overnight in 100% ethanol. After rehydration, brains were washed five times in PBS with 0.1% Triton X-100 for 15 min at room temperature. Subsequently, they were incubated in probe hybridization buffer at 37 °C on a rotator for 1.5 h and incubated in probe hybridization buffer and relevant probes overnight. Probes (*HR38* with HCR amplifier B2-488 and *eyeless* with HCR amplifier B3-647) were washed with probe wash buffer five times for 15 min and five times for 15 min in 5× SSC with 0.1% Triton X-100 at room temperature. Then, brains were incubated for 30 min at room temperature in amplification buffer and overnight in heat-shocked amplification probes diluted in the same buffer at room temperature on a rotator. After incubation, brains were washed five times for 15 min in 5× SSC with 0.1% Triton X-100 and finally incubated in VECTASHIELD at 4 °C overnight before mounting and imaging with a ZEISS Airyscan 880 confocal microscope. Images were analyzed with ImageJ v.1.53t.

### Whole-mount brain staining

After dissection in S2 medium, fly brains were fixed in 4% PFA for 30 min. Then, brains were washed in PBS with 0.5% Triton X-100 two times for 15 min. Subsequently, brains were incubated in SNAP-tag at a dilution of 1:1,000 in PBS with 0.3% Triton X-100 for 30 min and a further 30 min with the addition of the Halo-tag at the same dilution. Lastly, brains were washed in PBS with 0.5% Triton X-100 two times for 15 min and incubated in VECTASHIELD overnight at 4 °C. Brains were mounted in VECTASHIELD and imaged on a ZEISS Airyscan 880 confocal microscope.

### Reporting summary

Further information on research design is available in the Nature Portfolio Reporting Summary linked to this article.

## Online content

Any methods, additional references, Nature Portfolio reporting summaries, source data, extended data, supplementary information, acknowledgements, peer review information; details of author contributions and competing interests; and statements of data and code availability are available at 10.1038/s41593-023-01549-4.

### Supplementary information

Supplementary Information Supplementary Fig. 1.

Reporting Summary

Supplementary Tables 1–7.

### Source data

Source Data Fig. 1 Meta-data of ethoscope recordings of flies used for 10x processing. Source Data Fig. 8 Meta-data of ethoscope recordings of flies used for the sleep rebound experiments. Source Data Extended Data Fig. 1 Meta-data of ethoscope recordings of flies used in the DGRP screen. Source Data Extended Data Fig. 7 Meta-data of ethoscope recordings of flies used for the FISH experiments.

Source Data Fig. 6 Maximum projection confocal images.

Source Data Extended Data Fig. 7 Maximum projection confocal images.

## Data Availability

The scRNA-seq data have been deposited in the Gene Expression Omnibus (GEO) under accession no. GSE221239. Publicly available data used in this study are available under the following GEO accession nos.: Davie et al.^14^ (GSE107451); Ma et al.^22^ (GSE157504). Gene expression across all cell populations are visualized at https://scope.aertslab.org/#/Fly_Brain_Sleep/Fly_Brain_Sleep%2FFly_Sleep.loom/gene. Transcript expression plots for all correlates and clusters are available at https://joana-dopp.shinyapps.io/Fly_Sleep_Single_Cell_v1/. Source data are provided with this paper.

## Code availability

The custom code associated with this study is available at https://github.com/shaliulab/Single_Cell_Sleep.
