# Supplementary material for: Single-cell transcriptomics reveals that glial cells integrate homeostatic and circadian processes to drive sleep–wake cycles
Source: Nat Neurosci. 2024 Jan 23;27(2):359–72. doi: 10.1038/s41593-023-01549-4 (PMC10849968; doi:10.1038/s41593-023-01549-4)
Supplement: Supplementary file 1 — Supplementary Fig. 1. [file 41593_2023_1549_MOESM1_ESM.pdf]

nature neuroscience

Resource https://doi.org/10.1038/s41593-023-01549-4

# Single-cell transcriptomics reveals that glial cells integrate homeostatic and circadian processes to drive sleep–wake cycles

In the format provided by the authors and unedited

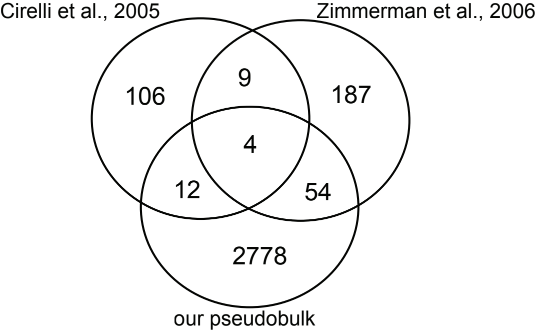


**Supplementary Figure 1. Bulk transcriptomics is insufficient to capture molecular sleep/wake correlates.**
Comparing sleep/wakefulness correlates across all cells combined found in this study with two previous bulk microarray studies (Cirelli et al. 2005, Zimmerman et al. 2006), that similarly profiled differences between sleep and wakefulness conditions, shows that the overlap of significant genes is minimal between all three studies.
