# Supplementary material for: Single-cell transcriptomics reveals that glial cells integrate homeostatic and circadian processes to drive sleep–wake cycles
Source: Nat Neurosci. 2024 Jan 23;27(2):359–72. doi: 10.1038/s41593-023-01549-4 (PMC10849968; doi:10.1038/s41593-023-01549-4)
Supplement: Supplementary file 2 — Reporting Summary [file 41593_2023_1549_MOESM2_ESM.pdf]

Corresponding author(s): Dr. Sha Liu

updated by author(s): Dec 4, 2023

Reporting Summary

Nature Portfolio wishes to improve the reproducibility of the work that we publish. This form provides structure for consistency and transparency in reporting. For further information on Nature Portfolio policies, see our Editorial Policies and the Editorial Policy Checklist.

Statistics

For all statistical analyses, confirm that the following items are present in the figure legend, table legend, main text, or Methods section.

n/a	Confirmed	
☐	☒	The exact sample size (*n*) for each experimental group/condition, given as a discrete number and unit of measurement
☒	☐	A statement on whether measurements were taken from distinct samples or whether the same sample was measured repeatedly
☐	☒	The statistical test(s) used AND whether they are one- or two-sided<br>*Only common tests should be described solely by name; describe more complex techniques in the Methods section.*
☒	☐	A description of all covariates tested
☐	☒	A description of any assumptions or corrections, such as tests of normality and adjustment for multiple comparisons
☐	☒	A full description of the statistical parameters including central tendency (e.g. means) or other basic estimates (e.g. regression coefficient) AND variation (e.g. standard deviation) or associated estimates of uncertainty (e.g. confidence intervals)
☐	☒	For null hypothesis testing, the test statistic (e.g. *F*, *t*, *r*) with confidence intervals, effect sizes, degrees of freedom and *P* value noted<br>*Give P values as exact values whenever suitable.*
☒	☐	For Bayesian analysis, information on the choice of priors and Markov chain Monte Carlo settings
☒	☐	For hierarchical and complex designs, identification of the appropriate level for tests and full reporting of outcomes
☒	☐	Estimates of effect sizes (e.g. Cohen's *d*, Pearson's *r*), indicating how they were calculated

Our web collection on statistics for biologists contains articles on many of the points above.

Software and code

Policy information about availability of computer code

Data collection

To collect sleep behaviour data, ethoscopes were used (Geissmann et al., 2017). To sequence cDNA libraries, 10x Genomics Chromium Single Cell 3' Kit (v3.1) NextGEM chemistry was used. To capture confocal images a Zeiss Airyscan 880 confocal microscope was used.

Data analysis

python=3.7.3 anndata=0.7.8 numpy=1.19.5 pandas=1.3.4 scanpy=1.4.4 seaborn=0.11.2 fastp=0.20.0 STAR=2.7.9a scikit-lean=1.0.2 interpret=0.2.7 pySCENIC=0.11.2 (https://github.com/aertslab/pySCENIC) vsn-pipelines=0.25.0 (https://github.com/vib-singlecell-nf/vsn-pipelines)
r-base=3.6.3 CellRanger=3.1.0 ggplot2=3.3.6 MetaCycle=1.2.0 shiny=1.7.3 tidyverse=1.3.2 gprofiler2=0.2.1 GOSemSim=1.20.0 rrvgo=1.6.0 rutils=0.99.2

ethoscope analysis: https://github.com/rethomics
Analysis pipelines of sleep drive template matching and EBM classifier can be found at https://github.com/shaliulab/Single_Cell_Sleep

ImageJ=1.53t http://imagej.nih.gov/ij

For manuscripts utilizing custom algorithms or software that are central to the research but not yet described in published literature, software must be made available to editors and reviewers. We strongly encourage code deposition in a community repository (e.g. GitHub). See the Nature Portfolio guidelines for submitting code & software for further information.

Data

Policy information about availability of data

All manuscripts must include a data availability statement. This statement should provide the following information, where applicable:

- Accession codes, unique identifiers, or web links for publicly available datasets
- A description of any restrictions on data availability
- For clinical datasets or third party data, please ensure that the statement adheres to our policy

The scRNA-seq data has been deposited in GEO under accession code GSE221239. Publicly available data utilized in this study are available under the following GEO accession codes: Davie et al. (2018): GSE107451; Ma, Przybylski et al. (2021): GSE157504. Gene expression across all cell populations are visualized at https://scope.aertslab.org/#/Fly_Brain_Sleep/Fly_Brain_Sleep%2FFly_Sleep.loom. Transcript expression plots for all correlates and clusters are available at https://joana-dopp.shinyapps.io/Fly_Sleep_Single_Cell/. Both links can be found at https://www.flysleeplab.com/scsleepbrain.

Human research participants

Policy information about studies involving human research participants and Sex and Gender in Research.

Reporting on sex and gender	not applicable
Population characteristics	not applicable
Recruitment	not applicable
Ethics oversight	not applicable

Note that full information on the approval of the study protocol must also be provided in the manuscript.

Field-specific reporting

Please select the one below that is the best fit for your research. If you are not sure, read the appropriate sections before making your selection.

☒ Life sciences ☐ Behavioural & social sciences ☐ Ecological, evolutionary & environmental sciences

For a reference copy of the document with all sections, see nature.com/documents/nr-reporting-summary-flat.pdf

Life sciences study design

All studies must disclose on these points even when the disclosure is negative.

Sample size	In 7 runs, a total of 282 flies were collected across 11 different behavioural conditions and four circadian times, outlined in Supplementary Table 1. With around 40 brains per run, we aimed to acquire 10K cells per experimental run (see Supplementary Table 7), since previous work (Davie et al., 2018) showed that with this number the main cell types would be represented.
Data exclusions	10x data were filtered according to the Data Processing steps outlined in the text, including doublet removal, removal of cells expressing less than 200 genes or 20-30% UMIs assigned to mitochondrial genes.
Replication	10x experiments were repeated 7 times with variations in combination of genotype and condition. Validation of main findings was performed by FISH (at least 4 brains per condition, 5-7 days old male flies with consistent results) and sleep rebound behaviour (at least 4 independent experiments per genotype, 4-7 days old mated females).
Randomization	For each of seven technical replicates, genotype and conditions were randomly shuffled.
Blinding	Investigators were not blind to group assignment. Each group of flies was sampled at different points of behavioural state and circadian time point. The investigator needed to know the group assignment to select the animals to be sampled.

Reporting for specific materials, systems and methods

March 2021

We require information from authors about some types of materials, experimental systems and methods used in many studies. Here, indicate whether each material, system or method listed is relevant to your study. If you are not sure if a list item applies to your research, read the appropriate section before selecting a response.

Materials & experimental systems

n/a	Involved in the study
☒	☐ Antibodies
☒	☐ Eukaryotic cell lines
☒	☐ Palaeontology and archaeology
☐	☒ Animals and other organisms
☒	☐ Clinical data
☒	☐ Dual use research of concern

Methods

n/a	Involved in the study
☒	☐ ChIP-seq
☒	☐ Flow cytometry
☒	☐ MRI-based neuroimaging

Animals and other research organisms

Policy information about studies involving animals; ARRIVE guidelines recommended for reporting animal research, and Sex and Gender in Research

Laboratory animals	DGRP (88, 287, 303, 313, 359, 379, 441, 646, 892 and 908), repo-Gal4>iso31 (n=58), repo-Gal4>UAS-Cas9.P2 (n=30), repo-Gal4>UAS-sgRNA-vri (n=41), repo-Gal4>UAS-sgRNA-tim (n=26), repo-Gal4>UAS-sgRNA-cry (n=41), repo-Gal4>UAS-sgRNA-vri; UAS-Cas9.P2 (n=30), repo-Gal4>UAS-sgRNA-tim;UAS-Cas9.P2 (n=47), repo-Gal4>UAS-sgRNA-cry;UAS-Cas9.P2 (n=35), repo-Gal4>UAS-Cyc-delta (n=48). All flies were 4-9 days old
Wild animals	NO wild animals involved in this study
Reporting on sex	single-cell RNA sequencing was performed on male flies only to avoid sex-based batch effects. Validation experiments were performed on both male and mated female.
Field-collected samples	NO field-collected samples involved in this study
Ethics oversight	Ethical approval is not required for Drosophila research in the EU.

Note that full information on the approval of the study protocol must also be provided in the manuscript.
