# Supplementary figures and images for: Single-cell transcriptomics reveals that glial cells integrate homeostatic and circadian processes to drive sleep–wake cycles
Source: Nat Neurosci. 2024 Jan 23;27(2):359–72. doi: 10.1038/s41593-023-01549-4 (PMC10849968; doi:10.1038/s41593-023-01549-4)

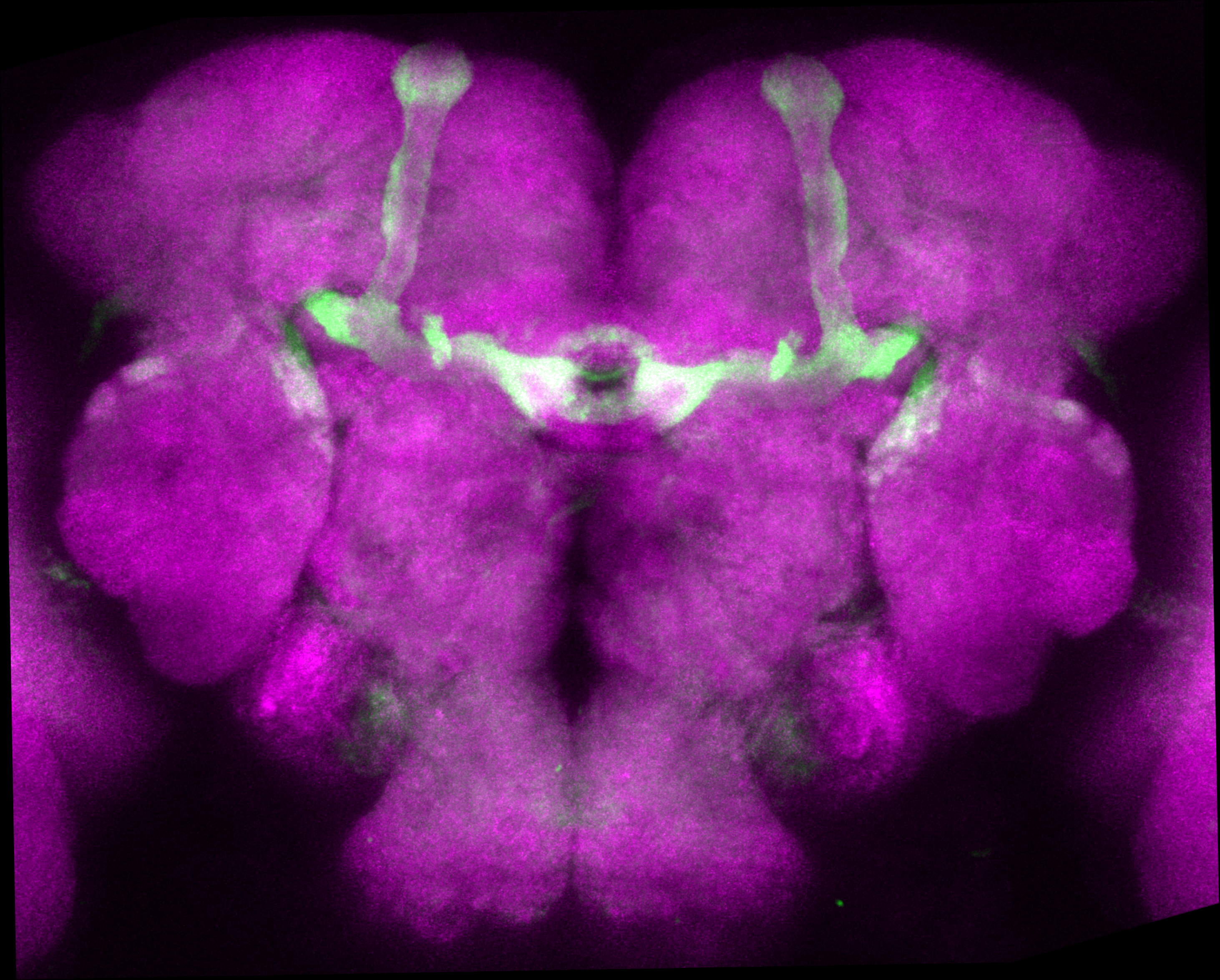

Supplement: Supplementary file 5 — Maximum projection confocal images. [file 41593_2023_1549_MOESM5_ESM.zip › 5ht1a.tif]

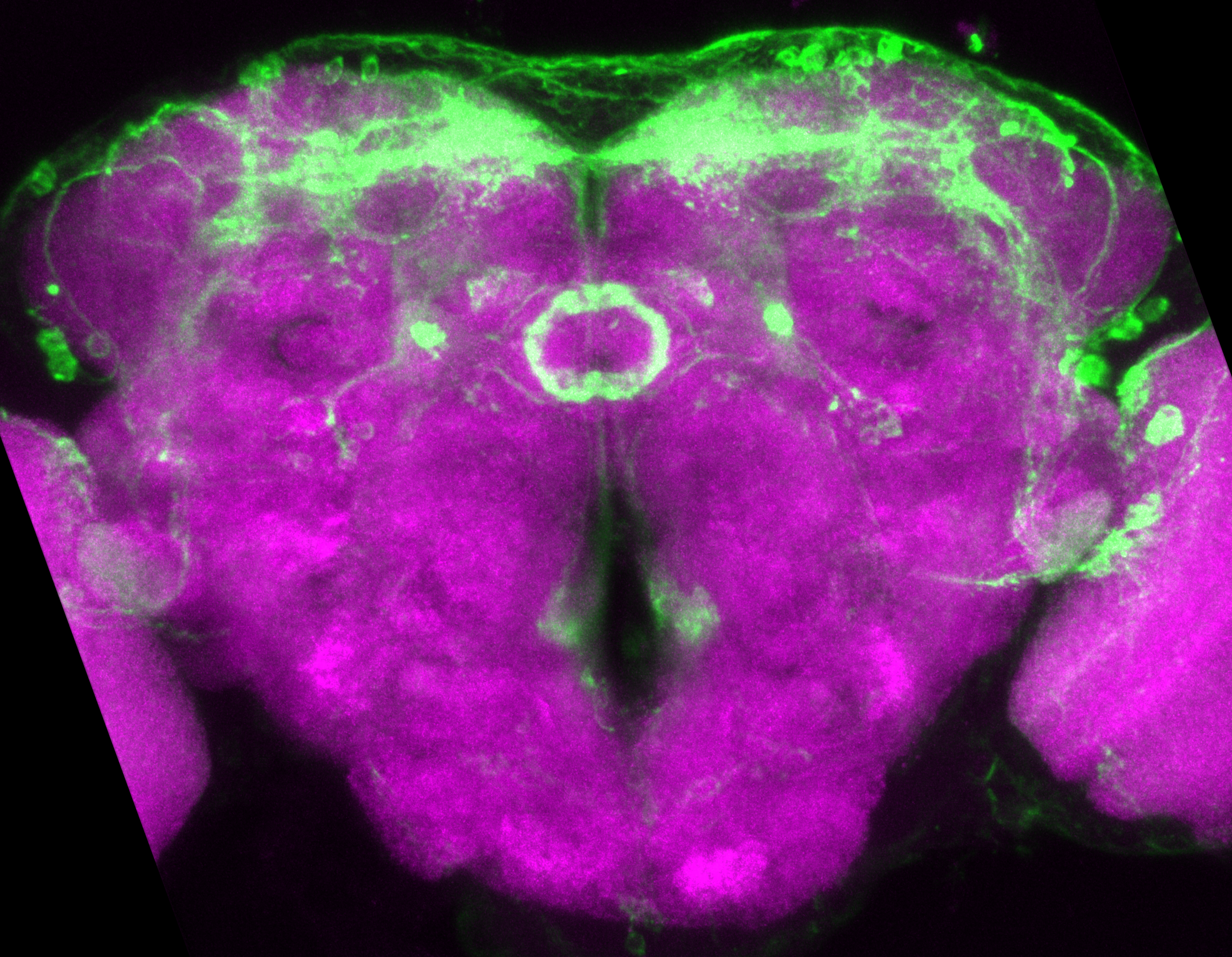

Supplement: Supplementary file 5 — Maximum projection confocal images. [file 41593_2023_1549_MOESM5_ESM.zip › cry.tif]

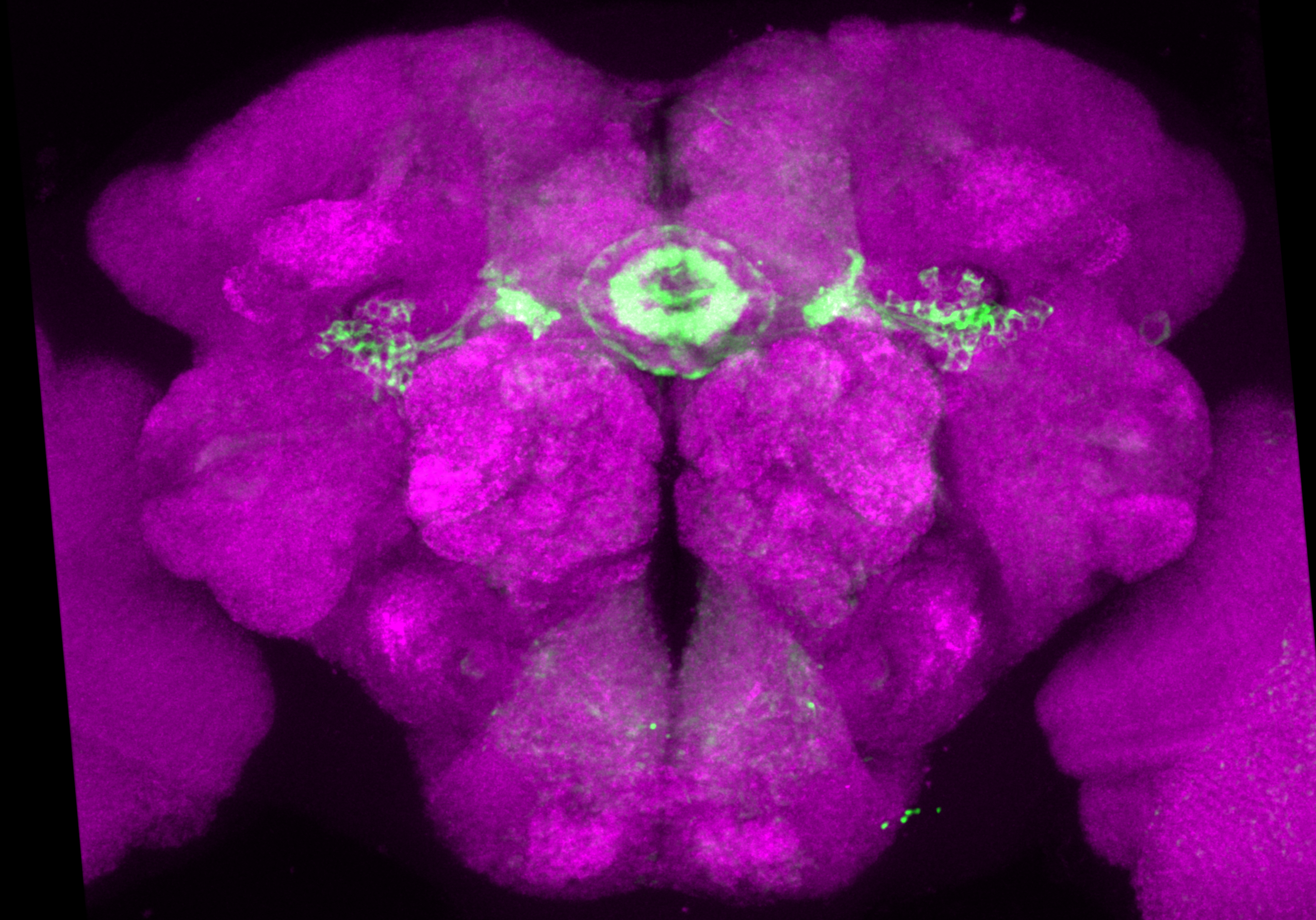

Supplement: Supplementary file 5 — Maximum projection confocal images. [file 41593_2023_1549_MOESM5_ESM.zip › 5ht2b.tif]

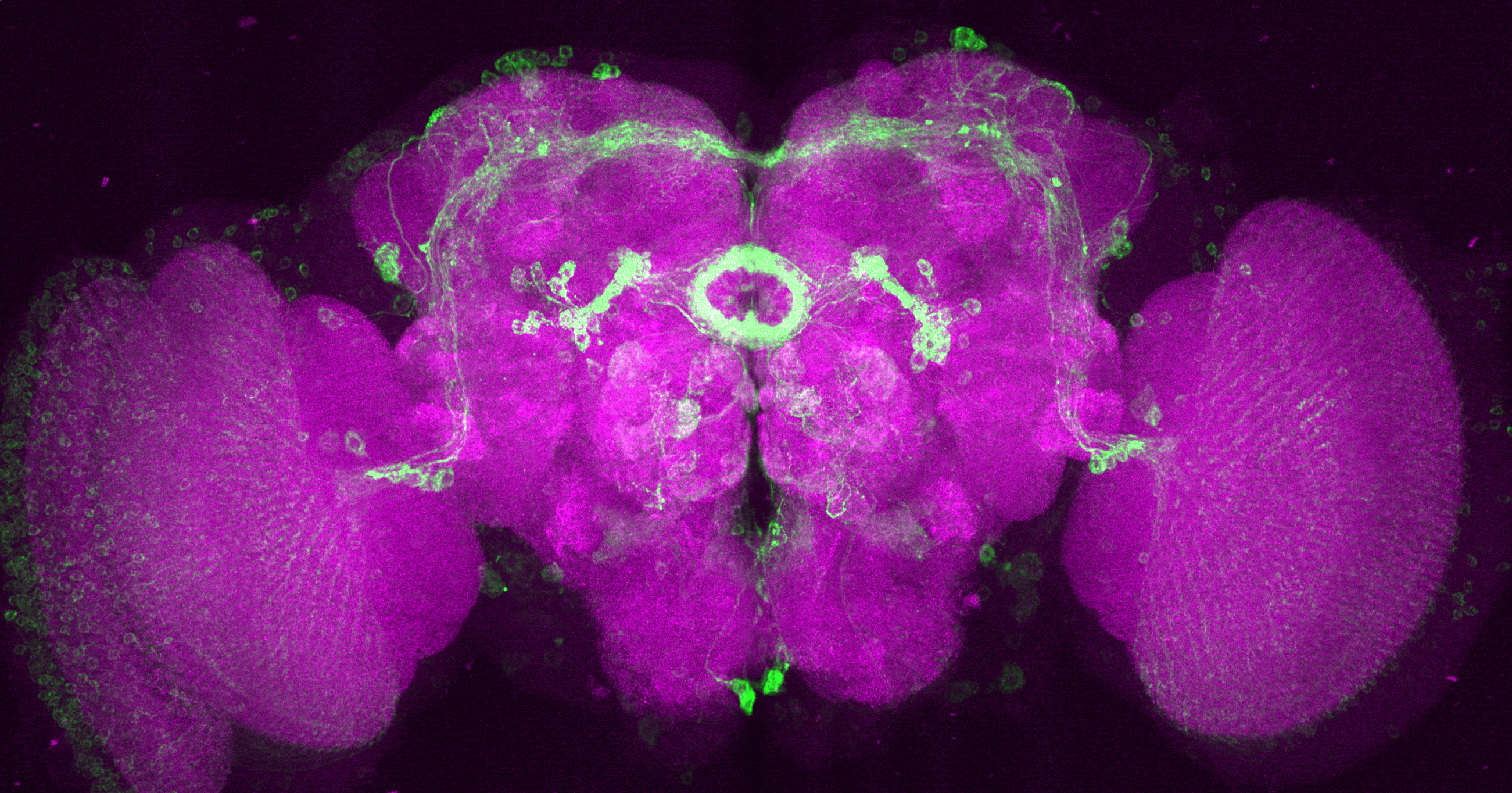

Supplement: Supplementary file 5 — Maximum projection confocal images. [file 41593_2023_1549_MOESM5_ESM.zip › pdfr.tif]

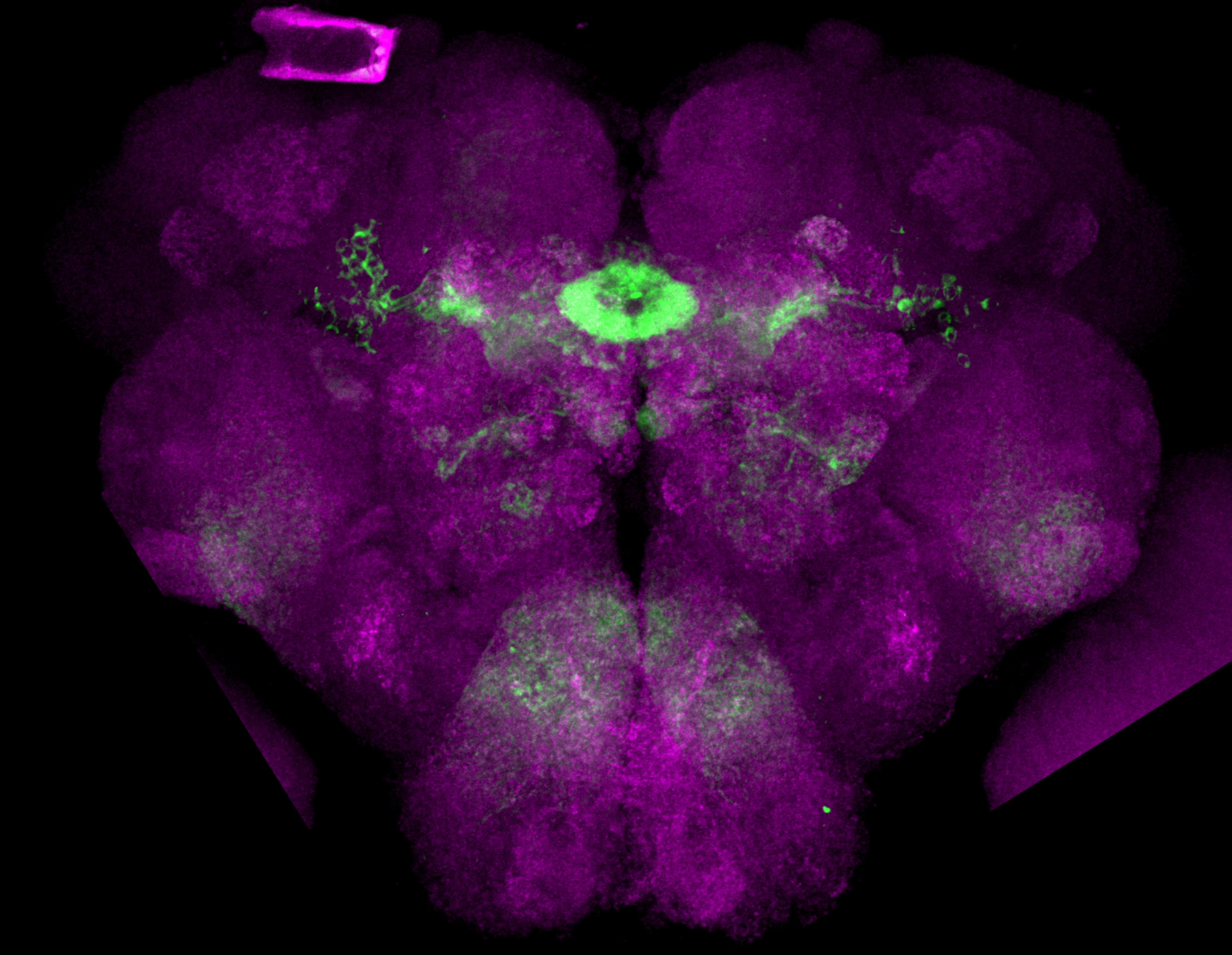

Supplement: Supplementary file 5 — Maximum projection confocal images. [file 41593_2023_1549_MOESM5_ESM.zip › 5ht7.tif]

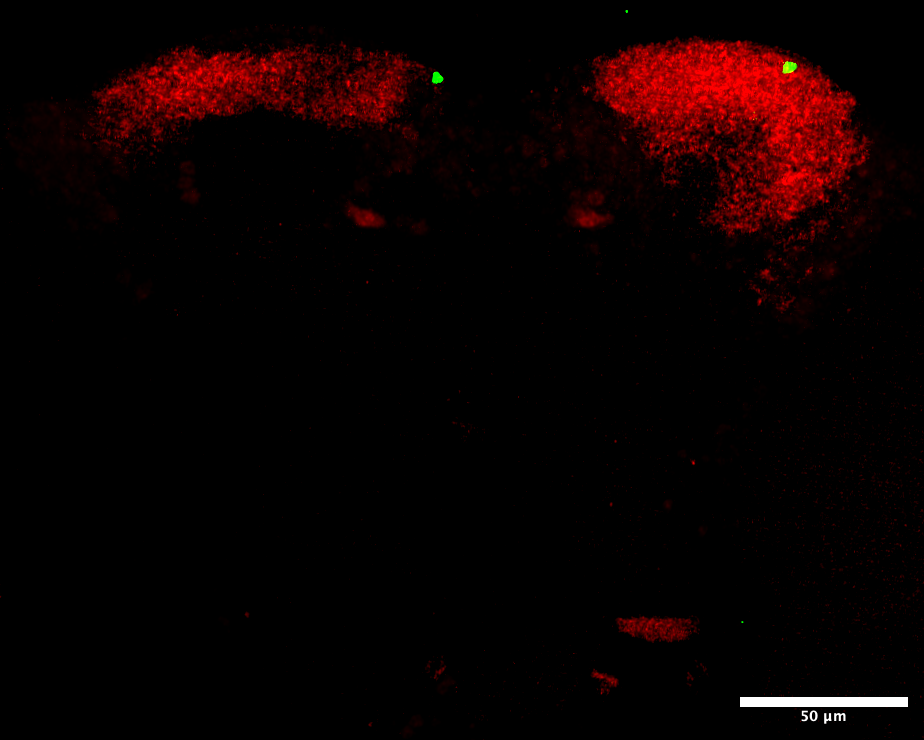

Supplement: Supplementary file 6 — Maximum projection confocal images. [file 41593_2023_1549_MOESM6_ESM.zip › sleep.tif]

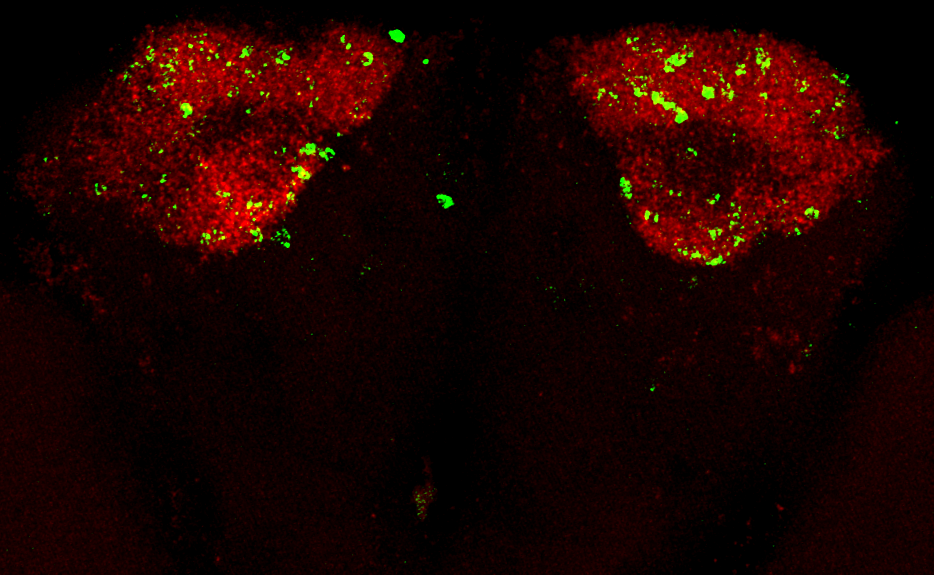

Supplement: Supplementary file 6 — Maximum projection confocal images. [file 41593_2023_1549_MOESM6_ESM.zip › sleepdeprivation.tif]
